# Salvigenin Suppresses Hepatocellular Carcinoma Glycolysis and Chemoresistance Through Inactivating the PI3K/AKT/GSK-3β Pathway

**DOI:** 10.1007/s12010-023-04511-z

**Published:** 2023-05-02

**Authors:** Hui Shao, Jingyan Chen, Ali Li, Lili Ma, Yongzhi Tang, Huazhong Chen, Yongping Chen, Junyan Liu

**Affiliations:** 1 Department of Infection, Zhejiang Taizhou Hospital Affiliated to Wenzhou Medical University, Linhai City, 317000 Zhejiang Province China; 2 Department of Infectious and Liver Diseases, the First Affiliated Hospital of Wenzhou Medical University, Wenzhou, 325000 Zhejiang Province China

**Keywords:** Salvigenin, Hepatocellular carcinoma, PI3K, Glycolysis, Chemoresistance, Apoptosis

## Abstract

Salvigenin is a Trimethoxylated Flavone enriched in *Scutellariae Barbatae Herba* and *Scutellariae Radix* and is demonstrated to have anti-tumor properties in colon cancer. Notwithstanding, the function and mechanism of Salvigenin in hepatocellular carcinoma (HCC) are less well studied. Different doses of Salvigenin were taken to treat HCC cells. Cell viability, colony formation ability, cell migration, invasion, apoptosis, glucose uptake, and lactate production levels were detected. As shown by the data, Salvigenin concentration dependently dampened HCC cell proliferation, migration, and invasion, weakened glycolysis by abating glucose uptake and lactate generation, and suppressed the profiles of glycolytic enzymes. Moreover, Salvigenin strengthened HCC cells’ sensitivity to 5-fluorouracil (5-FU) and attenuated HCC 5-FU-resistant cells’ resistance to 5-FU. Through network pharmacological analysis, we found Salvigenin potentially regulates PI3K/AKT pathway. As shown by the data, Salvigenin repressed the phosphorylated levels of PI3K, AKT, and GSK-3β. The PI3K activator 740Y-P induced PI3K/AKT/GSK-3β pathway activation and promotive effects in HCC cells. However, Salvigenin substantially weakened 740Y-P-mediated effects. *In-vivo* assay revealed that Salvigenin hampered the growth and promoted apoptosis of HCC cells in nude mice. Collectively, Salvigenin impedes the aerobic glycolysis and 5-FU chemoresistance of HCC cells by dampening the PI3K/AKT/GSK-3β pathway.

**Supplementary Information:**

The online version contains supplementary material available at 10.1007/s12010-023-04511-z.

## Introduction

Hepatocellular carcinoma (HCC), a primary malignancy of hepatocytes, has high-level invasiveness [1]. In the early stage, HCC patients show no evident symptoms, leading to delayed diagnosis at the earliest time and missing the opportunity of receiving curative treatment (such as liver resection and liver transplantation) [2, 3]. Furthermore, subsequent to liver resection treatment, HCC patients have recurrence rates as high as 50% and 70% at 3 years and 5 years [4]. For advanced HCC, doxorubicin was commonly used in the treatment of HCC. Single-agent doxorubicin gained 79% objective response rates at the initial phase but demonstrated only limited efficacy (<20% clinical responses) without significant survival benefit [5]. Of note, the 5-year survival rate of HCC patients in China only attains 12% [6]. Over the past years, traditional Chinese medicine (TCM) has been extensively applied to cancer patients. It has been substantiated to alleviate chemotherapy toxicity and ameliorate patients’ prognosis [7, 8]. Thus, a probe into the mechanism of HCC development is critical to developing efficacious treatment strategies.

Metabolic reprogramming, particularly aerobic glycolysis (also referred to as the Warburg effect), was first detected in HCC [9]. As HCC progresses, the energy metabolism of cancer cells converts to aerobic glycolysis, offering an enabling environment for cancer cell growth [10]. Aerobic glycolysis augments glucose uptake and lactate generation under normoxic circumstances, hence driving HCC cells’ proliferation, migration, invasion, angiogenesis, immune escape, and chemoresistance [11]. As reported, suppressing HCC cell aerobic glycolysis can prompt cancer cells to be sensitive to chemotherapy drugs again [12].

*Scutellariae Barbatae Herba* (SBH), a dried whole plant of *Scutellaria Barbata D. Don*, is referred to as Ban-Zhi-Lian in traditional Chinese medicine (TCM). SBH, equipped with anti-cancer, anti-oxidant, and anti-angiogenic activities, enjoys a history of thousands of years in China [13–15]. The latest studies have denoted that SBH can be employed for treating primary liver cancer and lung cancer [16, 17]. *Scutellariae Radix*, the root of *Scutellaria Baicalensis*, boasts anti-inflammatory and anti-cancer functions [18]. *Scutellariae Radix* exerts a remarkable anti-cancer function in liver cancer [19]. Salvigenin, a Trimethoxylated Flavone derived from *Scutellariae Barbatae Herba* and *Scutellariae Radix*, is reported to show the dual activities of reducing lipid and boosting mitochondrial functions [20]. Furthermore, Salvigenin also has good anti-tumor activities. For instance, Salvigenin can elicit cycle arrest in colon cancer cells and foster cancer cell apoptosis [21]. Salvigenin strengthens the anti-tumor immunity of the breast cancer mouse model and hinders tumor growth [22].

Network pharmacology is an emerging field in terms of the pharmacological research of modern Chinese medicine. It reveals the underlying molecular mechanisms of traditional Chinese medicine potency via the establishment and visualization of the “drug-target-disease” interactive network [23, 24]. Here, we discussed the function and molecular mechanism of Salvigenin in the context of HCC. We discovered that Salvigenin dampened HCC cell proliferation, migration, invasion, glycolysis, and chemoresistance. Moreover, we conducted network pharmacological analysis and forecasted the targets of Salvigenin and HCC via databases. The common targets between Salvigenin and HCC were found by establishing a network and then subjected to the Gene Ontology (GO) analysis and the Kyoto Encyclopedia of Genes and Genomes (KEGG) pathway analysis. We also unveiled the major biological processes, molecular functions, and relevant pathways of Salvigenin fighting HCC. As a result, PI3K/AKT/GSK-3β might be the molecular mechanism of Salvigenin in HCC. At last, we uncovered that PI3K/AKT/GSK-3β pathway activation was inextricably associated with the malignant development of HCC by consulting the literature [25, 26]. However, PI3K/AKT/GSK-3β pathway inhibition hindered the aerobic glycolysis of HCC cells and boosted their apoptosis [27]. Given these findings, we conjectured that Salvigenin might exert its cancer-suppressing function in HCC via the PI3K/AKT/GSK-3β pathway.

## Materials and Methods

### Network Pharmacological Analysis

We acquired the targets related to hepatocellular carcinoma (HCC) from the GeneCards (https://www.genecards.org/) database and obtained Salvigenin-associated targets from SwissTarget (http://www.swisstargetprediction.ch/) [28]. The Isomeric SMILES of Salvigenin was downloaded from the PubChem database (https://pubchem.ncbi.nlm.nih.gov/). Venn’s diagram online tool (https://bioinfogp.cnb.csic.es/tools/venny/index.html) was adopted to analyze the crossing targets of the disease and drug. The Cytoscape software (version 3.9.1) was harnessed to visualize the drug-target network diagram. We uploaded the common targets to the String database (https://cn.string-db.org/cgi/input.pl) to produce the protein-protein interaction (PPI) network diagram. The DAVID database (https://david.ncifcrf.gov/home.jsp) was introduced for the GO and KEGG enrichment analyses of the common targets.

### Cell Culture

The Human HCC cell lines (HCCLM3, Huh7, MHCC97H, MHCC97L, HepG2, and Hep3B) were ordered from American Type Culture Collection. The cells were cultured with an RPMI1640 medium (12633012, ThermoFisher Scientific, China) filled with 10% fetal bovine serum (FBS, 10100147, ThermoFisher Scientific, China) and 1% penicillin/streptomycin in an incubator (37 °C, 5% CO_2_, humid air).

### Cell Viability and Toxic Detection

The CCK-8 kit (C0037, Beyotime Biotechnology, Shanghai, China) was adopted to examine cell viability. We inoculated HCC cells into 96-well plates (density: 1×10^3^ cells/well) for overnight culture. Predicated on prior studies, we treated the cells with Salvigenin (0 μM, 25 μM, 50 μM, 100 μM, 200 μM, 400 μM, 800 μM) or 5-FU (0 μM, 25 μM, 50 μM, 100 μM, 200 μM, 400 μM, 800 μM) for 24 h. Next, 10 μL of CCK-8 solution was administered to each well for 2 h of further incubation (37 °C, 5% CO_2_). At the end of the culture, a microplate reader (SpectraMax Mini, Molecular Devices) was utilized to gauge the absorbance value at 450 nm. Santa Cruz Biotechnology, Inc. (https://www.scbt.com/zh/p/salvigenin-19103-54-9) was the supplier of Salvigenin (CAS number: 19103-54-9). Cell death was assessed using the Calcein/PI Cell Viability/Cytotoxicity Assay Kit (C2015M, Beyotime Biotechnology, Shanghai, China). Huh7 and HepG2 cells were seeded into 96-well plates (density: 1×10^3^ cells/well) for overnight culture. Followed by Salvigenin (25, 50, 100μM) treatment, Calcein AM/PI working solution was added into each well (100 per well), and the plates were put in a 37 °C incubator for 30 min. The Calcein AM/PI fluorescence signals were observed and taken using a microscope (Olympus, Tokyo, Japan).

### Colony Formation Assay

Huh7 and HepG2 cells were seeded onto 60-mm culture dishes with a density of 1×10^3^ cells/well. Salvigenin (100 μM), 740Y-P (50 μg/mL) or 5-FU (100 μM) for two days. After the medium was removed, the cells were cultured in a fresh culture medium without the above drugs. The medium was exchanged every 3 days; 10 days later, the colonies were fixed using 70% methanol for 30 min. Then, they were stained in 0.5% crystal violet at room temperature (RT) for 30 min. A light microscope (FluoView ™ FV1000, Olympus, Japan) was utilized for counting the number of cell colonies.

### Reverse Transcription-Polymerase Chain Reaction (RT-PCR)

TRIzol reagent (Thermo Fisher Scientific) was taken to extract total RNA from the cells. Nano Drop 2000 (ThermoFisher Scientific, Inc.) was adopted to measure the purity and concentration of the total RNA. As per the manufacturer’s recommendations, the RevertAid First Strand cDNA Synthesis Kit (Thermo Fisher Scientific, Inc.) was exploited to reverse-transcribe 1 μg of the total RNA into cDNA, which was utilized as the template to amplify the target gene and reference gene GAPDH. SYBR GreenPCR (MedChemExpress) was introduced for PCR. Thermal cycling was implemented with these conditions: 3 min of initial degeneration at 95 °C, 10 s of denaturation at 95 °C, 30 s of annealing at 60 °C, and 30 s of extension at 60 °C (40 cycles in total). The 2^−ΔΔCT^ approach was employed to calculate the relative expression of the target gene (GAPDH as the internal parameter). The primers were designed via Primer3 (https://primer3.ut.ee/). The primer sequences are exhibited in Table 1.

**Table 1:** The primer sequences for qRT-PCR

Name	Forward primers (5′-3′)	Reverse primers (5′-3′)
HK2	GACCAACTTCCGTGTGCTTT	TCCATGAAGTTAGCCAGGCA
PFK1	TGTGTTCATCGTGGAGACCA	GTCATGGCACTTCTCGTTCC
PKM2	ACTTGGCGGCAACAGAATTT	TCCAGGAATGTGTCAGCCAT
GAPDH	CCAAGGAGTAAGACCCCTGG	TGGTTGAGCACAGGGTACTT

### Western Blot

We harvested the tumor tissues from the nude mice and HCC cells treated with varying doses of Salvigenin (25–100 μM) and/or 740Y-P (50 μg/mL). RIPA lysis buffer (P0013C, Beyotime Biotechnology, Shanghai, China) supplemented with a mixture of protease and phosphatase inhibitors (P1046, Beyotime Biotechnology, Shanghai, China) was adopted to extract total protein from the cells and tumor tissues, respectively. The Bicinchoninic Acid (BCA) protein quantification kit (P0012S, Beyotime Biotechnology, Shanghai, China) was harnessed for protein quantification. Then, 50 μg of the total protein samples from each group were isolated through 10% sodium dodecyl sulfate polyacrylamide gel electrophoresis (SDS-PAGE) and moved onto polyvinylidene difluoride (PVDF) membranes (FFP24, Beyotime Biotechnology, Shanghai, China); 5% skimmed milk was given to seal the membranes at RT for 2 h. Next, the membranes were incubated with primary antibodies (Anti-PI3K (ab191606, Abcam, 1:1000), Anti-p-PI3K (PA5-104853, Thermo Fisher,1:1000), Anti-AKT (ab235958, Abcam, 1:1000), Anti-p-AKT (ab38449, Abcam, 1:1000), Anti-GSK-3β (ab32391, Abcam, 1:1000), Anti-p-GSK-3β (ab75814, Abcam, 1:1000), Anti-HK2 (ab2209847, Abcam, 1:1000), Anti-PFK1 (#22772, signalway antibody, 1:1000), Anti-PKM2 (ab150377, Abcam,1:1000), and Anti-β-actin (#5174, 1:1000, cell signal)) at 4 °C overnight. Later, the horseradish peroxidase (HRP)-conjugated goat anti-rabbit IgG secondary antibody (ab6721, Abcam, 1:5000) was added to the membranes for 2 h of incubation at RT. Color and image development was done with the use of ECL (P0018FS, Beyotime Biotechnology, Shanghai, China) in darkness. β-actin served as the internal parameter.

### Transwell Migration and Invasion Experiments

Transwell compartments (3244; pore size: 8μm; Corning) were placed in 24-well plates; 50 μL of diluted Matrigel (1:8; BD Biosciences) was added to the upper compartment in the invasion experiment instead of the migration assay. With the cells suspended in a medium without serum (1×10^5^ cells/mL), 200 μL of the cell suspension was given to the upper chamber, whereas 600 μl of a medium with 15% FBS was administered to the lower compartment. A sterile, thermostatic incubator was adopted to culture the cells for 24 h at 37 °C with 5% CO_2_. The compartments were taken out. The cells that failed in migration and invasion were wiped off using cotton swabs. Transwell membranes were immobilized in 4% paraformaldehyde for 10 min and dyed in 0.1% crystal violet. They were observed and photographed employing a microscope.

### Determination of Glucose Uptake and Lactate Production

The supernatant of the mediums of Huh7 and HepG2 cells was harvested. The floating cells and cell debris were removed through 5 min of centrifugation (1000 g, 4 °C). Then, the supernatant was harvested. As instructed by the manufacturer, the glucose uptake detection kit (ab65333, Abcam, USA) and the lactate production detection kit (ab65330, Abcam, USA) were taken to determine the levels of glucose uptake and lactate generation.

### Animal Experiment

Male BALB/c nude mice, 6–8 weeks of age and 20 ± 2 g in weight, were ordered from the Animal Experimental Center of Wenzhou Medical University. They were reared under specific-pathogen-free (SPF) conditions (relative humidity, 60% ± 5%; temperature, 22 ± 2 °C) with a 12-h light/dark cycle. They could access food and water at will. With the cell density adjusted to 4×10^6^ cells/mL, Huh7 cells were suspended in a serum-free medium and subcutaneously transfused into the right abdomen of the mice (0.1 mL each). In this way, a liver cancer xenograft model was built. A vernier caliper was taken to gauge the length (L) and width (W) of transplanted tumors every three days. As the tumor volume attained around 50 mm^3^, the animals were randomized to different groups (*n* = 5 per group). Salvigenin (5 μg/mouse/day, 10 μg/mouse/day) was intraperitoneally injected into the mice for treatment. Normal saline of the identical amount was given to the control group. The mouse tumor bodies were examined every week for 4 weeks running. *V* (mm^3^) = *L* × *W*^2^/2. When the experiment came to an end, the mice were sacrificed through the inhalation of excessive CO_2_. The tumors were separated and photographed. Their volume and weight were gauged. The tumors were harvested for the following experiments. All experiments, authorized by the Ethics Committee of Zhejiang Taizhou Hospital, affiliated with Wenzhou Medical University, were implemented in conformity with the *Guidelines for the Care and Use of Laboratory Animals* (NIH publications Nos. 80-23, revised in 1996).

### Immunohistochemistry (IHC)

The mouse tumor tissues were immobilized using 4% paraformaldehyde, dehydrated with alcohol of gradient concentrations, made transparent with xylene, embedded in paraffin, and severed into slices (4-μm thick). The sections were dewaxed and hydrated; 3% H_2_O_2_ was applied to block the activity of endogenous peroxidase. After antigen repair, the slices were sealed using 5% goat serum for 20 min, flushed in PBS three times, and then incubated along with Anti-Ki67 (ab15580, Abcam,1:200) antibody overnight at 4 °C. Afterward, the sections were incubated with the HRP-conjugated goat anti-rabbit IgG secondary antibody (ab6721, Abcam, 1:1000) for 2 h. Diaminobenzidine (DAB) was taken for color development. Hematoxylin was used for redyeing. The staining outcomes were observed employing a microscope (Olympus, Tokyo, Japan) after the slices were dehydrated, made transparently, and sealed by neutral gum.

### HE Staining

The paraffin slices of tumor tissues were dehydrated using gradient alcohol, washed in distilled water, dyed with the hematoxylin solution for 8 min, flushed in running water, and turned blue with 0.6% ammonia. Subsequently, the sections were stained in the eosin solution for 2 min, dehydrated, and made transparent with the use of xylene. Finally, they were dried and sealed with neutral gum. A microscope (Olympus, Tokyo, Japan) was utilized for observation and photography.

### Tissue Immunofluorescence

The paraffin slices were routinely dewaxed to water and subjected to antigen repair. After PBS washing, the sections were sealed using 5% bovine serum for 30 min. We added the primary antibodies Anti-PI3K (ab191606, Abcam, 1:250) and Anti-AKT (ab8805, Abcam, 1:200) to incubate them overnight at 4 °C. Next, the fluorescence-labeled goat anti-rabbit IgG secondary antibody (ab6939, Abcam, 1:2000) was applied for 1-h incubation at RT. DAPI was utilized for redyeing. An anti-fluorescence quenching agent was adopted to seal the sections. A fluorescence microscope (Olympus, Tokyo, Japan) was taken for observation and imaging.

### TdT-mediated DUTP Nick End Labeling (TUNEL) Staining

After the HCC cells were treated with Salvigenin (100 μM), 740Y-P (50 μg/mL), or 5-FU (100 μM), as mentioned before, the medium was removed. The cells were flushed in pre-cooled PBS, fixed with 4% polyformaldehyde for 60 min, and rinsed in PBS once. Later, an immunostaining detergent was applied for 2 min of incubation in an ice bath. The samples were incubated for an hour in darkness at 37 °C with 50 μL TUNEL solution. After being flushed in PBS three times, the slices were sealed using an antifade mounting medium (P0131, Beyotime Biotechnology, Shanghai, China) and observed by a fluorescence microscope (Olympus, Tokyo, Japan).

As per the instructions of the TUNEL apoptosis kit (C1090, Beyotime Biotechnology, Shanghai, China), the tumor slices were routinely dewaxed, hydrated, and flushed using PBS three times. The Protease K working solution (20 μg/mL) without any DNase (ST523, Beyotime Biotechnology, Shanghai, China) was added for 15 min of reaction at 37 °C. The slices were rinsed in PBS three times. Next, 50 μL TUNEL solution was added and evenly spread over the samples. After being sealed with an anti-fluorescence quenching agent, the slices were observed by a fluorescence microscope.

### Biochemical Analysis

The mouse blood samples were harvested. The levels of alanine aminotransferase (ALT), aspartate aminotransferase (AST), blood urea nitrogen (BUN), and serum creatinine (Scr) were through the Fuji Dri-Chem 3500i Biochemistry Analyzer (Fujifilm Ltd, Japan).

### Statistical Analysis

GraphPad Prism 8.0 (GraphPad Software, Inc.) was introduced for analyzing statistics. Measurement statistics were exhibited as mean ± standard deviation (SD). One-way ANOVA was taken for comparison among multiple groups, followed by post hoc Tukey’s test, while a *t*-test was implemented to compare data differences between two groups. *P* < 0.05 held statistical significance.

## Results

### Salvigenin Dampened HCC Cell Proliferation, Migration, and Invasion and Boosted Apoptosis

**Fig. 1.**
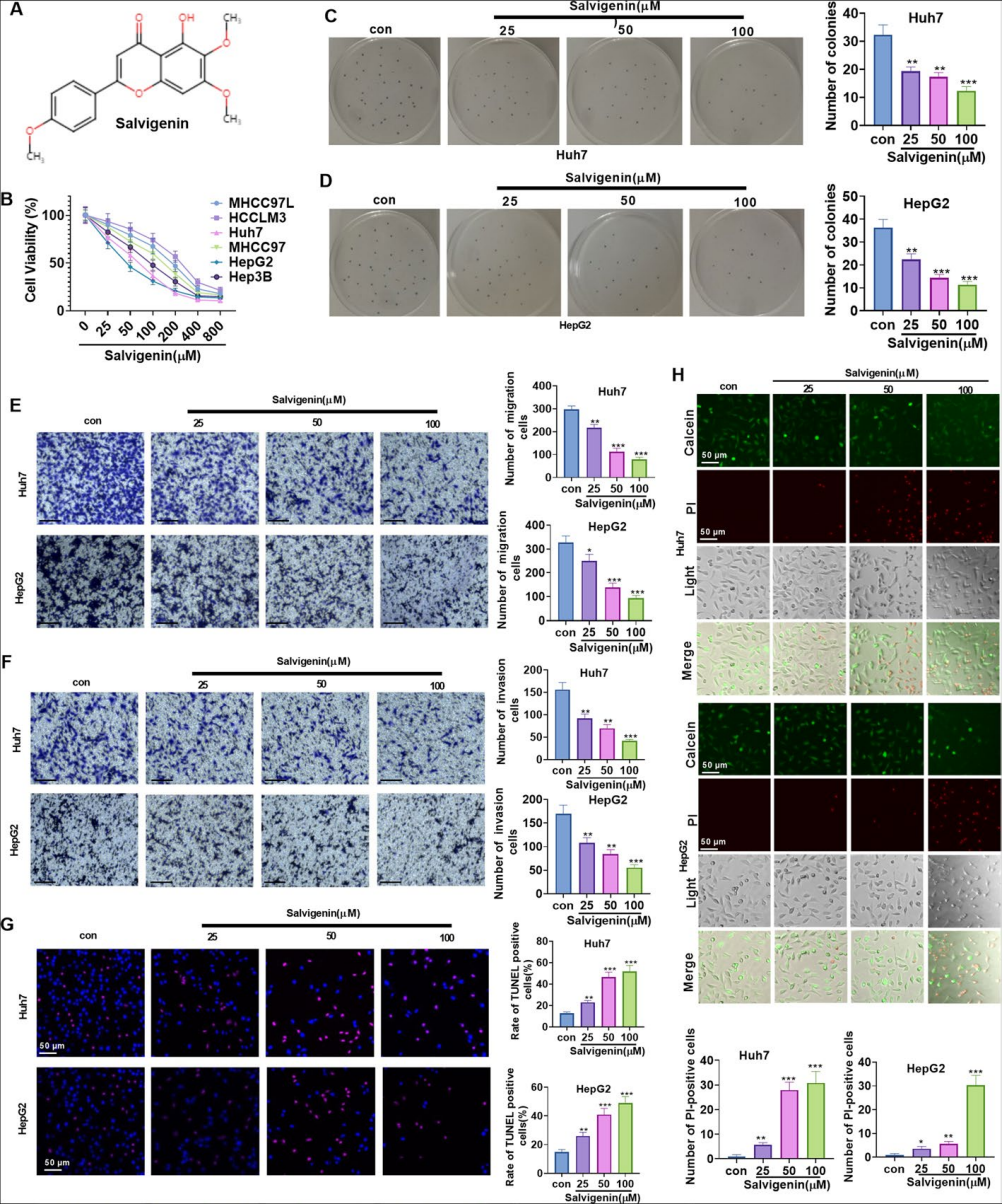
Salvigenin dampened HCC cell proliferation, migration, and invasion and boosted apoptosis. (**A**) The structural formula of Salvigenin. Salvigenin (0 μM, 25 μM, 50 μM, 100 μM, 200 μM, 400 μM, 800 μM) was applied to treat six types of HCC cells. (**B**) Salvigenin concentration dependently suppressed cell viability. Huh7 and HepG2 cells were the most sensitive to Salvigenin. Salvigenin (25 μM, 50 μM, 100 μM) was taken to treat Huh7 and HepG2 cells. (**C**) Colony formation assay. (**D**, **E**) Transwell monitored migration and invasion. (**F**, **G**) TUNEL examined apoptosis. (**H**) Cell death was determined using the Calcein/PI Cell Viability/Cytotoxicity Assay Kit. *P < 0.05, **P < 0.01, ***P < 0.001 (vs. the con group). *N* = 3

To investigate the pharmacological function of Salvigenin (Fig. 1A: the chemical formula of Salvigenin) in the context of HCC, we examined the influence of Salvigenin (0 μM, 25 μM, 50 μM, 100 μM, 200 μM, 400 μM, 800 μM) on six types of HCC cells. It transpired that Salvigenin suppressed cell viability in most HCC cells in a concentration-dependent pattern (Fig. 1B). We also adopted Salvigenin (25 μM, 50 μM, 100 μM) to treat Huh7 and HepG2 cells (which are most sensitive to Salvigenin) with a view to further studying its function in HCC. A colony formation assay was performed to measure colony-forming ability. Transwell monitored migration and invasion, while TUNEL detected apoptosis. The outcomes revealed that Salvigenin concentration dependently impeded colony formation (Fig. 1C), migration, and invasion (Fig. 1D, E) and bolstered apoptosis (Fig. 1F, G). Furthermore, we tested cell death using the Calcein/PI Cell Viability/Cytotoxicity Assay Kit. Our data showed that Salvigenin enhanced PI-positive cells and reduced Calcein expression in cells (Fig. 1H). These discoveries denoted that Salvigenin repressed the malignant biological behaviors of HCC cells.

### Salvigenin Impeded HCC Cells’ Aerobic Glycolysis

**Fig. 2:**
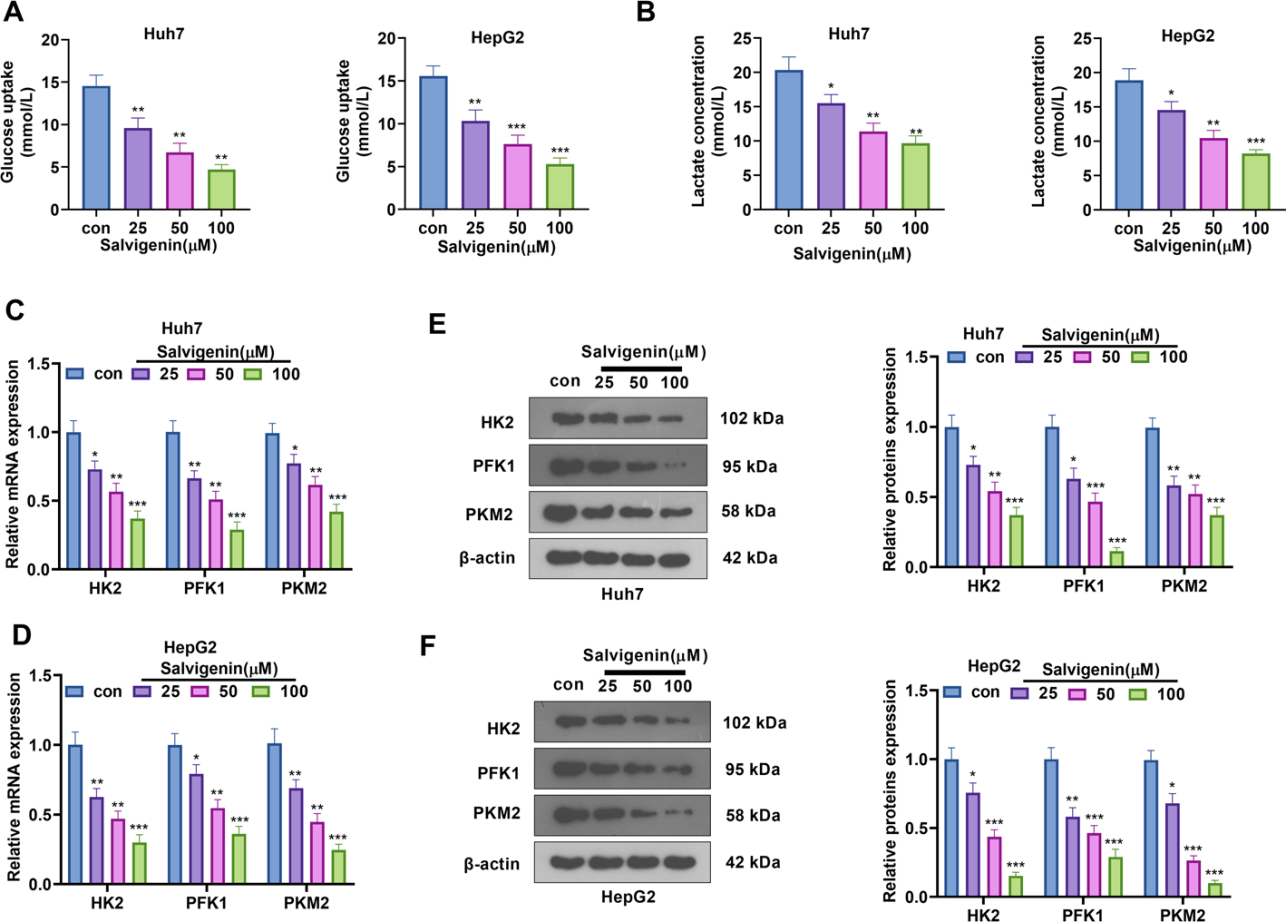
Salvigenin repressed HCC cell aerobic glycolysis. Salvigenin (25 μM, 50 μM, 100 μM) was utilized to treat Huh7 and HepG2 cells. (**A**) The level of glucose uptake. (**B**) The level of lactate production. (**C**, **D**) RT-PCR measured the relative levels of glycolytic genes (HK2, PFK1, PKM2). (**E**, **F**) Western blot determined the profiles of glycolytic enzymes. *P < 0.05, **P < 0.01, ***P < 0.001 (vs. the con group). *N* = 3

Aerobic glycolysis, a symbol of cancer metabolism, features glucose uptake and lactate generation. This prompted us to probe the influence of Salvigenin on the aerobic glycolysis of HCC cells. Corresponding kits were taken to examine the levels of glucose uptake and lactate generation in Huh7 and HepG2 cells. As a result, Salvigenin vigorously attenuated glucose uptake and lactate production (*P* < 0.05, Fig. 2A, B). RT-PCR and western blot verified the profiles of glycolytic rate-limiting enzymes (HK2, PFK1, PKM2). Our statistics reflected that Salvigenin concentration dependently restrained their expressions (*P* < 0.05, Fig. 2C). Given these discoveries, Salvigenin repressed the aerobic glycolysis of HCC cells.

### Salvigenin Blunted HCC Drug-resistant Cells’ Resistance to 5-FU

**Fig. 3:**
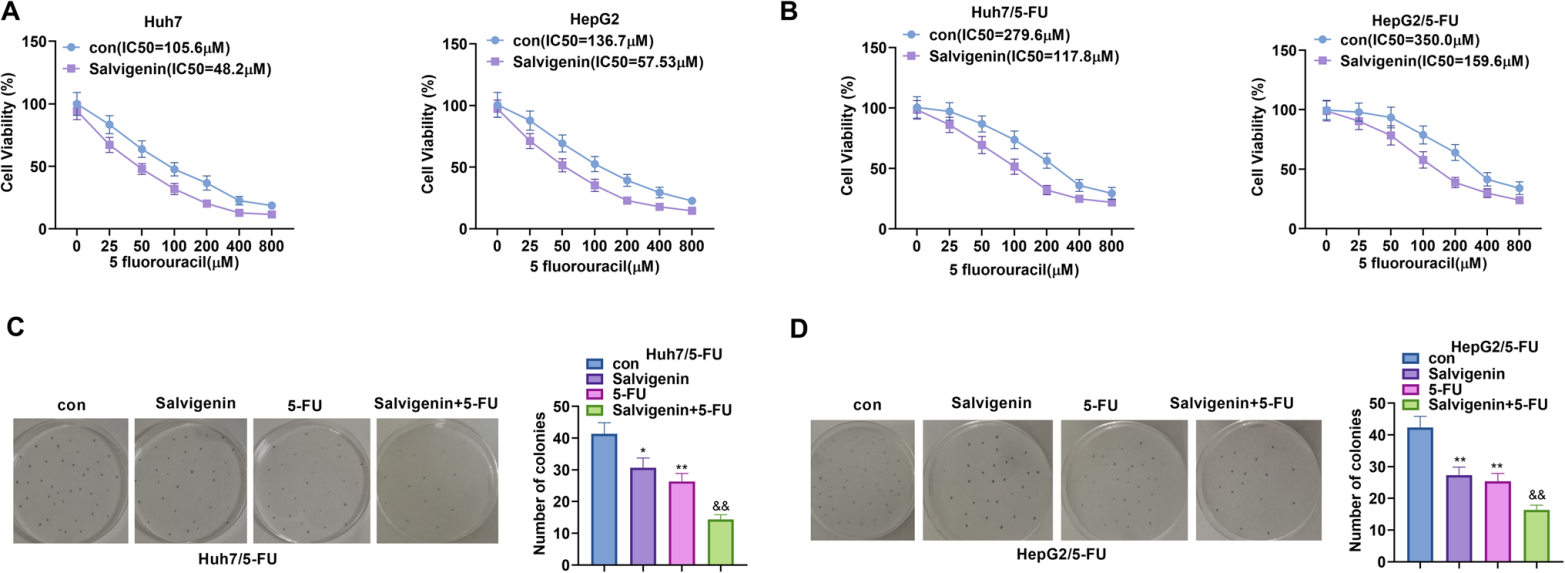
Salvigenin weakened HCC drug-resistant cells’ chemoresistance. Salvigenin (100 μM) and 5-FU (0 μM, 25 μM, 50 μM, 100 μM, 200 μM, 400 μM, 800 μM) were taken to treat Huh7 and HepG2 cells. (**A**) CCK-8 gauged cell viability. The IC50 value was calculated. The 5-FU-resistant Huh7 (Huh7/5-FU) and HepG2 (HepG2/5-FU) cell lines were built. (**B**) CCK-8 determined cell viability. The IC50 value was calculated. The drug-resistant cell lines were treated with Salvigenin and/or 5-FU (100 μM). (**C**) Colony formation assay monitored cell colony formation. *P < 0.05, **P < 0.01, ***P < 0.001 (vs. the con group). *N* = 3

Salvigenin (100 μM) and 5-FU (0 μM, 25 μM, 50 μM, 100 μM, 200 μM, 400 μM, 800 μM) were applied to treat Huh7 and HepG2 cells for the purpose of studying the influence of Salvigenin on HCC cells’ chemoresistance. CCK-8 tested cell viability, with the IC50 value calculated. The outcomes displayed that Salvigenin dramatically fortified HCC cells’ sensitivity to 5-FU (versus the con group) (*P*< 0 .05, Fig. 3A): the 5-FU-resistant Huh7 (Huh7/5-FU) and HepG2 (HepG2/5-FU) cell lines. We assessed the impact of Salvigenin on HCC cells’ resistance to 5-FU. Statistics demonstrated that Salvigenin lowered the IC50 value of 5-FU in HCC drug-resistant cell lines (Fig. 3B). To confirm the influence of Salvigenin on HCC drug-resistant cells, we used Salvigenin and/or 5-FU (100 μM) to treat the drug-resistant cells and implemented colony formation assay. It turned out that as opposed to the single use of 5-FU, its combination with Salvigenin vigorously abated the number of cell colonies (Fig. 3D). Our observation established that Salvigenin elevated HCC cells’ sensitivity to chemotherapy and attenuated HCC drug-resistant cells’ chemoresistance to 5-FU.

### Network Pharmacological Analysis of Salvigenin Target-hepatocellular Carcinoma Target Interaction

**Fig. 4:**
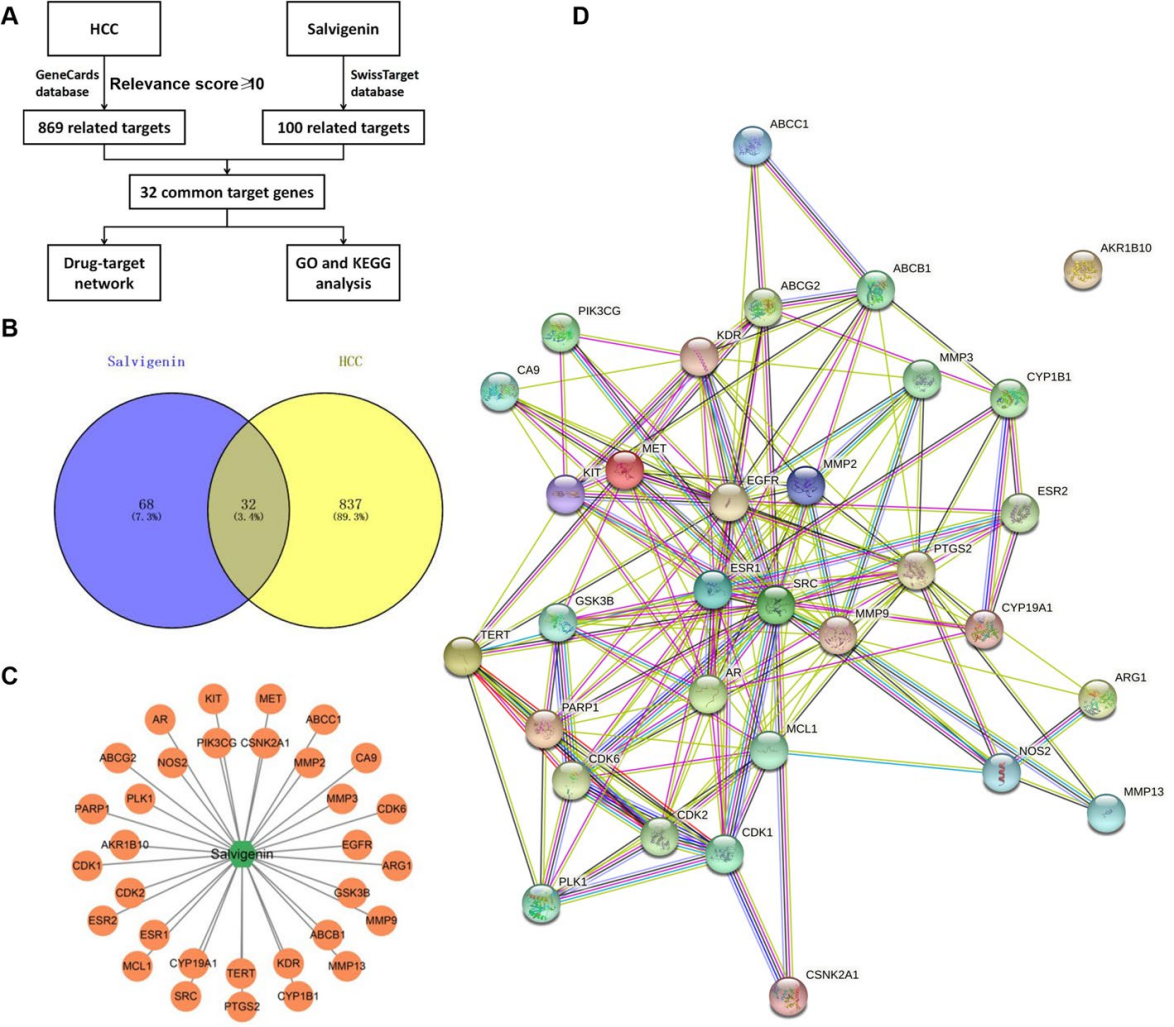
Network pharmacological analysis of Salvigenin target-HCC target interaction. (**A**) The flow chart of network pharmacological analysis. (**B**) Venn’s diagram displayed 32 common targets between HCC and Salvigenin. (**C**) The common targets of HCC and Salvigenin were utilized to establish the Compounds-Target Network diagram via the Cytoscape software (version 3.9.1). (**D**) The common targets of HCC and Salvigenin were uploaded to the String database (https://cn.string-db.org/cgi/input.pl). We selected Homo Sapiens to generate the PPI network diagram

To delve into the mechanism of Salvigenin treating HCC, we conducted a network pharmacological analysis. The analysis process is detailed in Fig. 4A. We acquired 869 HCC-associated targets (relevance score ≥ 10) from the GeneCards database (https://www.genecards.org/). The SMILES number of Salvigenin obtained from the PubChem database (https://pubchem.ncbi.nlm.nih.gov/) was inputted into the SwissTarget database (http://www.swisstargetprediction.ch/). We selected Homo Sapiens and downloaded 100 Salvigenin-concerned targets. The targets of the disease and drug were uploaded to Venn’s diagram online tool (https://bioinfogp.cnb.csic.es/tools/venny/index.html), and we obtained 32 common targets (Fig. 4B). The 32 targets were regarded as the underlying targets of Salvigenin fighting HCC. The Cytoscape software (version 3.9.1) was introduced for the construction of the Compounds-Target Network diagram (Fig. 4C). Next, the common targets were uploaded to the String database (https://cn.string-db.org/cgi/input.pl). We selected Homo Sapiens to generate the PPI network diagram (Fig. 4D).

### Salvigenin Potentially Modulated PI3K/AKT/GSK-3β in HCC

**Fig. 5:**
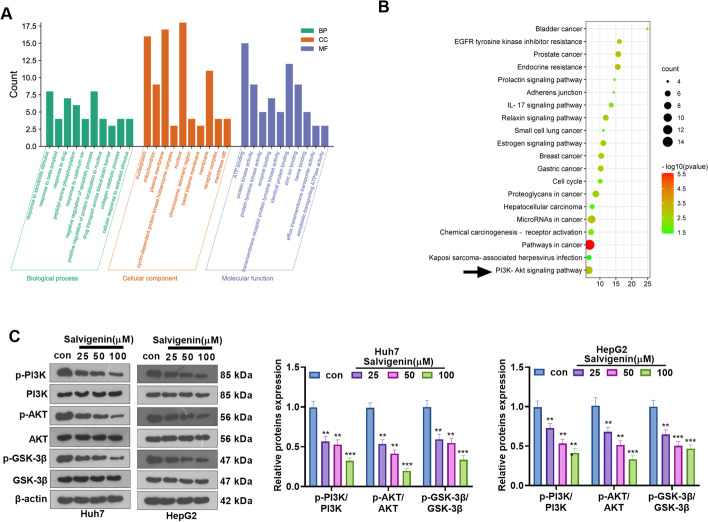
Salvigenin potentially modulated PI3K/AKT/GSK-3β in HCC. (**A**, **B**) The common targets were subjected to GO and KEGG enrichment analyses with the help of the DAVID database (https://david.ncifcrf.gov/home.jsp). The Weishengxin online drawing tool was adopted to visualize the top 20 signaling pathways and the top 10 terms of biological process (BP), cellular component (CC), and molecular function (MF). (**C**) Western blot verified the profile of PI3K/AKT/GSK-3β. *P < 0.05, **P < 0.01, ***P < 0.001 (vs. the con group). *N* = 3

To understand the mechanism of Salvigenin influencing the putative targets of HCC, we implemented the GO and KEGG enrichment analyses of the common targets with the help of the DAVID database (https://david.ncifcrf.gov/home.jsp) and visualized the top 20 signaling pathways and the top 10 terms of biological process (BP), cellular component (CC), and molecular function (MF) (Fig. 5A, B) through the Weishengxin online drawing tool. Our outcomes denoted that these common targets were enriched in the PI3K/AKT pathway. Interestingly, the GEPIA (http://gepia.cancer-pku.cn/) and The Human Protein Atlas (HTTPS://www.proteinatlas.org/) databases revealed that the expressions of PIK3CA, AKT1, and GSK3B in HCC tissues were enhanced (compared with those of normal liver tissues) (Supplementary Fig. 1A–G). The overall survival (OS) and disease-free survival (RFS) rates of patients with high PIK3CA, AKT1, and GSK3B expressions were lower than those of patients with low PIK3CA, AKT1, and GSK3B expressions (Supplementary Fig. 2A–C). To substantiate the mechanism of Salvigenin in HCC analyzed and predicted by network pharmacological analysis, we treated HCC cells (Huh7, HepG2) with Salvigenin (25 μM, 50 μM, 100 μM). Western blot ascertained the profile of PI3K/AKT/GSK-3β. It turned out that Salvigenin concentration dependently dampened PI3K, AKT, and GSK-3β phosphorylation (Fig. 5C, D). These discoveries established that Salvigenin might exert an anti-cancer function in HCC primarily by suppressing the PI3K/AKT/GSK-3β pathway.

### PI3K/AKT/GSK-3β Pathway Activation Weakened the Anti-cancer Function of Salvigenin

**Fig. 6:**
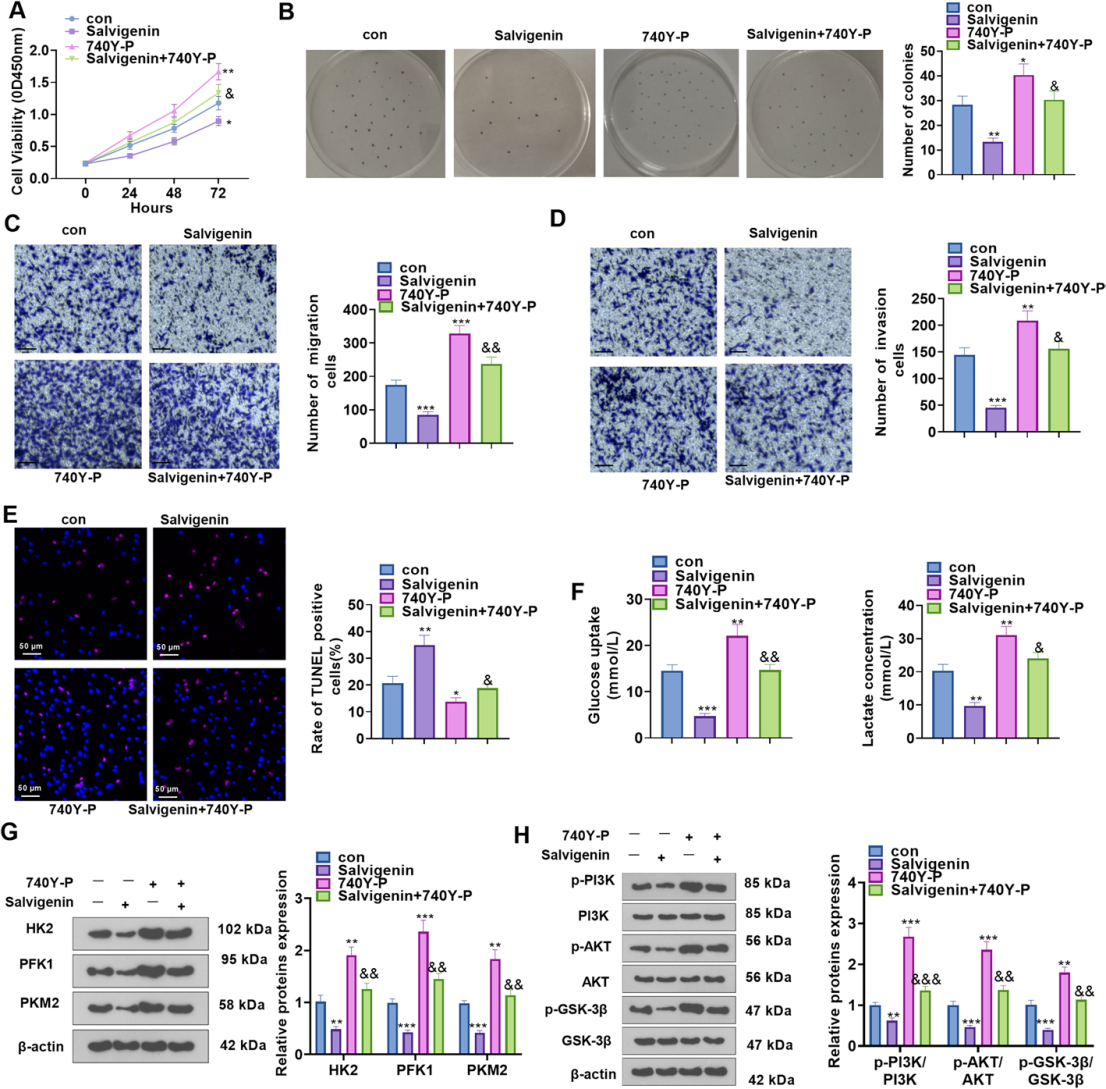
PI3K/AKT/GSK-3β pathway activation blunted the anti-cancer function of Salvigenin. Huh7 cells were treated using Salvigenin (100 μM) and/or 740Y-P (50 μg/mL, the PI3K agonist). (**A**) CCK-8 examined cell viability. (**B**) Colony formation assay measured cell colony formation. (**C**, **D**) Transwell assay monitored migration and invasion. (**E**) TUNEL assay checked apoptosis. (**F**) Glucose uptake and lactate generation levels were detected. (**G**, **H**) Western blot ascertained the profiles of glycolytic enzymes and the PI3K/AKT/GSK-3β pathway. *P < 0.05, **P < 0.01, ***P < 0.001 (vs. the con group), *&P* < 0.05, *&&P* < 0.01, *&&&P* < 0.001 (vs. the 740Y-P group). *N* = 3

To better understand the anti-cancer function of Salvigenin in HCC, we treated Huh7 cells with Salvigenin and/or 740Y-P (the PI3K activator). CCK-8, colony formation assay, Transwell migration and invasion experiments, and TUNEL were implemented. Figure 6A–D exhibited that by contrast to the Salvigenin group, 740Y-P distinctly heightened cell viability, augmented cell colony formation, bolstered cell migration and invasion, and attenuated cell apoptosis. Furthermore, the impact of 740Y-P on glycolysis was also confirmed. Corresponding kits were utilized to examine the levels of glucose uptake and lactate generation. Western blot checked the profiles of HK2, PFK1, and PKM2. As a result, 740Y-P notably fostered cell glycolysis (*P* < 0.05, Fig. 6E, F). At last, a western blot was carried out to determine the profile of the PI3K/AKT/GSK-3β pathway. It demonstrated that by contrast to the Salvigenin group, 740Y-P enhanced the profile of PI3K/AKT/GSK-3β phosphorylation (*P* < 0.05, Fig. 6G). On the evidence of the above data, PI3K/AKT/GSK-3β pathway activation weakened the anti-cancer function of Salvigenin in HCC.

### Salvigenin Repressed Tumor Growth in vivo

**Fig. 7:**
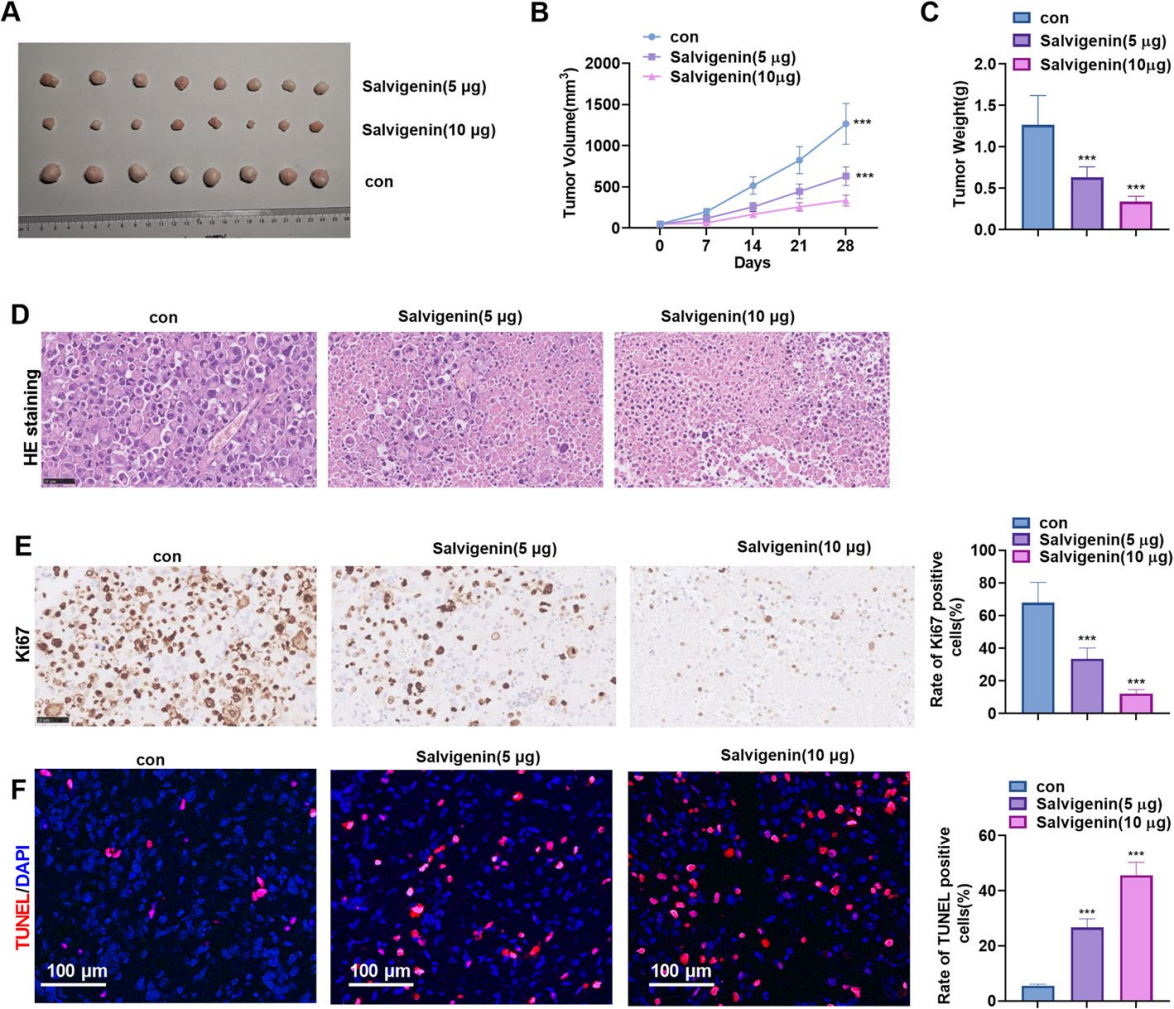
Salvigenin repressed tumor growth *in vivo.* A xenograft tumor model was built in nude mice with the use of Huh7 cells. Salvigenin (5 μg/mouse/day, 10 μg/mouse/day) was applied to treat the mice. (**A**) Tumors. (**B**) The volume of tumors. (**C**) The tumor weight. (**D**) HE staining monitored histopathological changes in the tumors. (**E**) IHC staining checked the positive rate of Ki67. (**F**) TUNEL assay determined apoptosis. *P < 0.05, **P < 0.01, ***P < 0.001 (vs. the con group). *N* = 5

A xenograft tumor model was built in nude mice with the use of Huh7 cells, and Salvigenin (5 μg/mouse/day, 10 μg/mouse/day) was taken to treat the mice. In this way, we probed the influence of Salvigenin on HCC cell growth *in vivo*. Salvigenin dose-dependently hampered tumor growth (Fig. 7A) and abated the tumor volume and weight (Fig. 7B, C). HE staining was adopted to monitor the histopathology of tumors (Fig. 7D). IHC staining measured the positive rate of Ki67, while TUNEL analyzed cell apoptosis. The outcomes manifested that Salvigenin lowered the positive rate of Ki67 and bolstered apoptosis (versus the con group) (Fig. 7E, F). For a probe into Salvigenin toxicity in tumor-bearing mice, ELISA was done to gauge the levels of ALT, AST, Scr, and BUN in the mouse serum subsequent to Salvigenin treatment. Following Salvigenin treatment, no remarkable alterations in the above indicators of hepatic and renal functions were detected (Supplementary Fig. 3A, B). HE staining reflected that Salvigenin exerted no evident pathological damage to the heart, liver, spleen, lung, and kidney tissues of the mice (Supplementary Fig. 3A, B). These discoveries signified that Salvigenin impeded HCC cell growth *in vivo* and had no substantial toxicity in the organs of tumor-bearing mice.

### Salvigenin Cramped Tumor Glycolysis and the PI3K/AKT/GSK-3β Pathway In Vivo

**Fig. 8:**
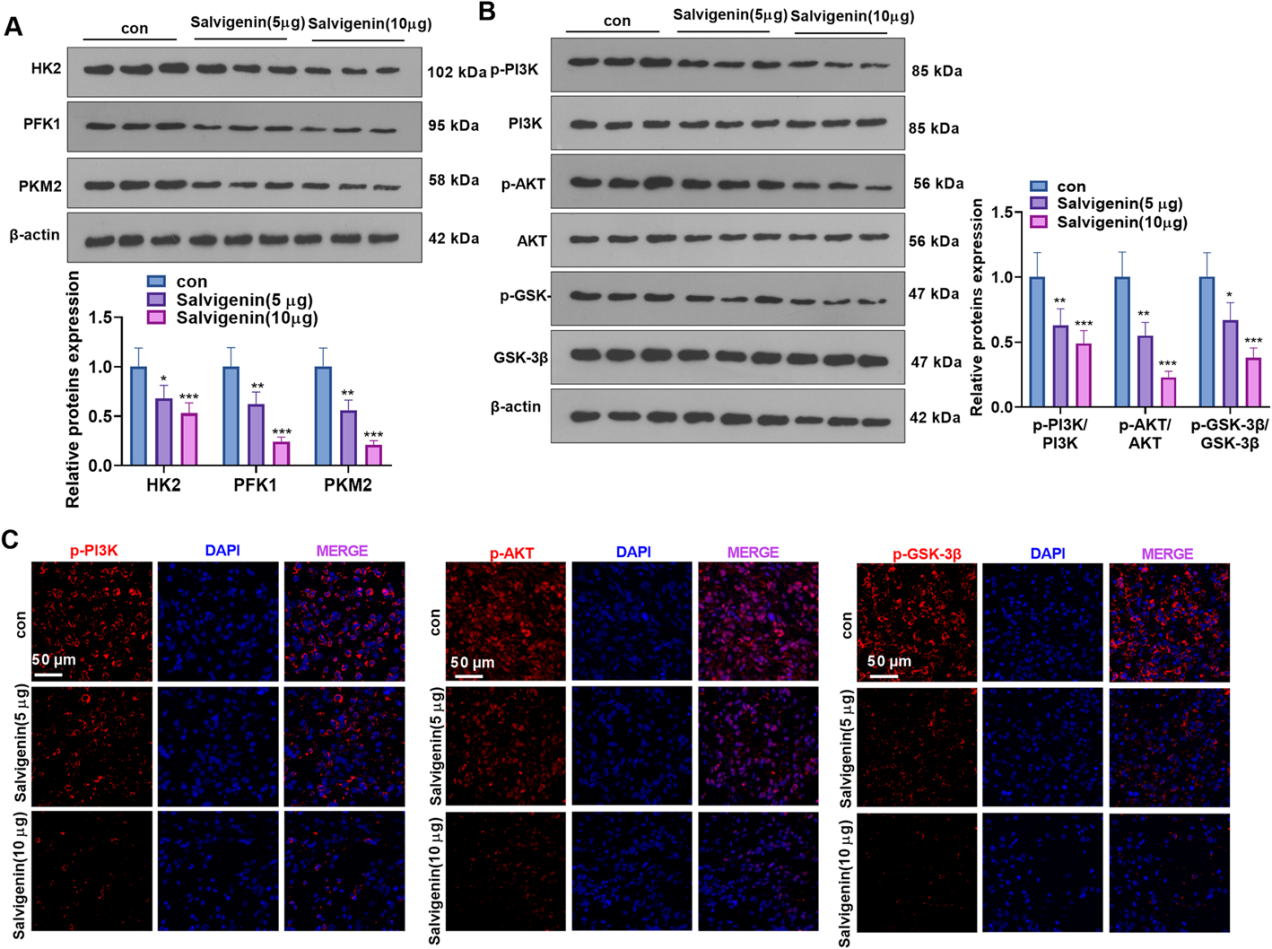
Salvigenin impeded tumor glycolysis and the PI3K/AKT/GSK-3β pathway *in vivo.* (**A**, **B**) Western blot confirmed the profiles of glycolytic enzymes and the PI3K/AKT/GSK-3β pathway. (**C**) Tissue immunofluorescence verified the profiles of PI3K and AKT. *P < 0.05, **P < 0.01, ***P < 0.001 (vs. the con group). *N* = 5

The profiles of HK2, PFK1, PKM2, and the PI3K/AKT/GSK-3β pathway were determined by western blot. The experimental statistics unveiled that Salvigenin restrained their expressions (Fig. 8A, B). Tissue immunofluorescence was also implemented to check the profile of PI3K/AKT signaling. Its outcomes displayed that Salvigenin weakened the fluorescence intensity of p-PI3K, p-AKT, and p-GSK-3β (Fig. 8C). Given these findings, Salvigenin suppressed tumor glycolysis and the PI3K/AKT/GSK-3β pathway *in vivo*.

## Discussion

HCC development, a sophisticated biological process, concerns multiple molecular and cellular signaling pathways [29]. Conventional treatment strategies are usually accompanied by high recurrence and metastasis rates. More efficacious therapies need to be developed for improving patients’ prognoses. On the back of the complexity of natural compounds, traditional Chinese medicine displays extensive pharmacological activities with various targets [30]. Our research confirmed that Salvigenin concentration dependently suppressed the malignant biological behaviors of HCC cells. Network pharmacological analysis was adopted to forecast the pharmacological mechanism of Salvigenin in HCC, which was substantiated by *in-vivo* and *in-vitro* experiments. Salvigenin exerted its cancer-suppressing function in the context of HCC by hampering the PI3K/AKT/GSK-3β signaling pathway.

**Fig. 9:**
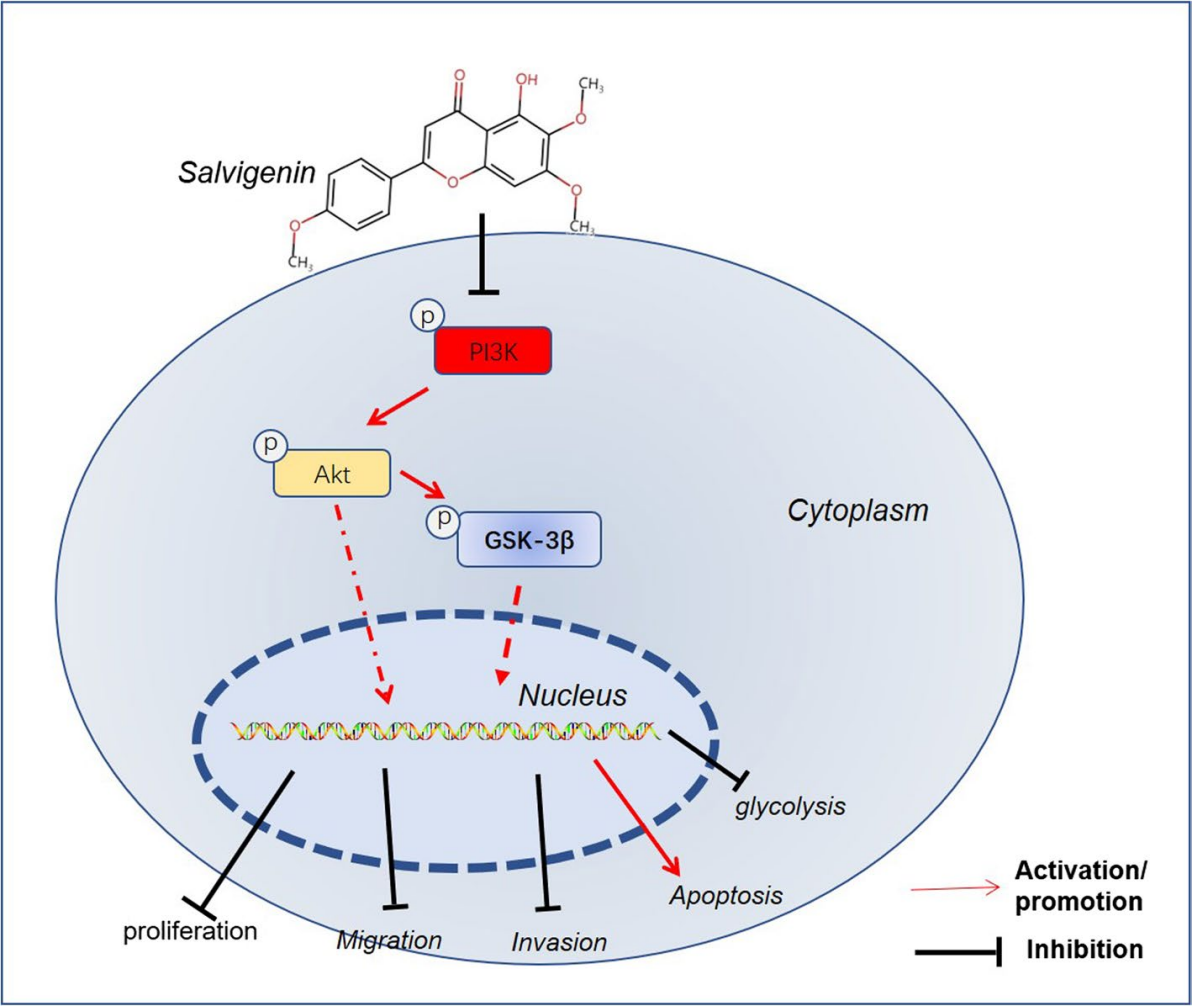
The mechanism diagram of Salvigenin in HCC

Network pharmacological analysis has indicated that Salvigenin may modulate cell proliferation and apoptosis so as to exert its anti-tumor function in HCC, which is a crucial factor contributing to HCC development [31]. Some studies have denoted that the signaling pathways pertaining to cell proliferation, migration, invasion, angiogenesis, and other processes are dysregulated in HCC development. These signaling pathways have become the targets for HCC treatment [32]. Here, network pharmacological analysis revealed that there were 32 underlying targets for Salvigenin to fight HCC. Moreover, multiple signaling pathways were discovered via GO and KEGG pathway enrichment analyses, including the PI3K/AKT/GSK-3β signaling pathway. Predicated on prior studies, we conjectured that Salvigenin might exert an anti-cancer function in HCC mainly via the PI3K/AKT/GSK-3β signaling pathway. Such a hypothesis prompted us to perform cell and animal experiments. As per the *in-vitro* outcomes, Salvigenin concentration dependently hampered HCC cell proliferation, migration, and invasion, elicited apoptosis, and weakened cell aerobic glycolysis and chemoresistance. *In-vivo* experiments unraveled that Salvigenin repressed the growth of tumors (Fig. 9).

Aerobic glycolysis is known as a marker of cancer metabolism. It has been demonstrated to be inextricably associated with HCC development [10]. Hexokinase (HK), phosphofructokinase (PFK), and pyruvate kinases (PKs) are three rate-limiting enzymes in the process of aerobic glycolysis. Alterations in their expressions influence HCC progression to a great extent. There are four sub-types of HK, namely HK1, HK2, HK3, and HK4. HK2 is a pivotal enzyme for the first step of catalyzing glycolysis. As reported, HK2 is dramatically up-regulated in HCC tissues, while HK2 knockdown dampens HCC cell aerobic glycolysis and tumor growth [33]. PFK1, a rate-limiting enzyme that partakes in the second step of glycolysis, catalyzes fructose 6-phosphate to generate fructose 1, 6-diphosphate. Metformin is reported to attenuate HCC cell glycolysis and repress cell proliferation by restraining PFK1 expression [34]. PK, a crucial enzyme for the last step of catalyzing glycolysis, produces pyruvic acid and ATP by transferring the phosphate group of phosphoenolpyruvic acid to ADP [35]. There are four isomers of PK to wit PKM1, PKM2, PKL, and PKR. PKM2 is the major type expressed and up-regulated in cancer [36]. In terms of HCC, targeted inhibition of PKM2 can antagonize glycolysis and re-sensitize sorafenib-resistant HCC cells to sorafenib [37]. Similarly, our study revealed that Salvigenin lessened glucose uptake and lactate generation, lowered the levels of HK2, PFK1, and PKM2, and abated 5-FU-resistant HCC cells’ resistance to 5-FU. These findings reflected that Salvigenin cramped the profiles of glycolytic rate-limiting enzymes (HK2, PFK1, and PKM2) to repress cell glycolysis and chemoresistance.

PI3K, an intracellular phosphatidylinositol kinase, has a critical function in cellular physiological processes (such as modulating cell proliferation, survival, protein synthesis, and glucose homeostasis) [38]. The PI3K pathway is initiated through the point mutation of the PIK3CA gene or the inactivation of the phosphatase and tensin homolog (PTEN) gene [39]. Mutations of PI3K pathway activation emerge in 30–50% of human cancers [40]. Activated PI3K produces PIP3 by catalyzing PIP2 phosphorylation. PIP3 is a significant lipid second messenger. It can combine and recruit cytoplasmic proteins with the pleckstrin-homology (PH) homologous structure to the cytoplasmic membrane, boosting the proteins to be activated [41]. Among these proteins containing the PH structure, the serine/threonine kinase AKT has drawn extensive attention. AKT, a pivotal downstream target of PI3K signaling, takes part in modulating multiple cellular pathways like proliferation, invasion, apoptosis, and angiogenesis. Targeting AKT is deemed a potential method for treating cancer [42]. The PI3K/AKT pathway is among the most common activated pathways during tumor occurrence and progression. PI3K/AKT pathway inhibition can attenuate HepG2 cell glycolysis [43]. Our research suggested that Salvigenin vigorously down-regulated the levels of PI3K, AKT, and GSK-3β phosphorylation.

Glycogen synthase kinase (GSK)-3β is a classical downstream target of the AKT pathway. Reportedly, GSK-3β has two phosphorylation sites (Ser9 and Tyr216). The phosphorylation of the Ser9 site can culminate in GSK-3β inactivation, whereas the phosphorylation of the Tyr216 site leads to GSK-3β activation [44]. The function of GSK-3β in tumors has been widely investigated, but the conclusions are still controversial. Some studies consider GSK-3β as a tumor-suppressing factor. For instance, GSK-3β expression is lowered in breast cancer tissues, while GSK-3β overexpression strengthens the inhibitory impact of Erastin on tumor growth [45]. Notwithstanding, GSK-3β is believed to have pro-cancer functions in other literature. For instance, GSK-3β expression is elevated in HCC tissues and is associated with the poor prognosis of patients [46]. Moreover, GSK-3β is a crucial enzyme that modulates glucose metabolism. Reportedly, GSK-3β partakes in HCC glycolysis. Suppressing GSK-3β activity can lower the levels of glucose uptake, lactate generation, and ATP in HCC cells and down-regulate the profiles of pivotal glycolytic enzymes [47]. Aligned with preceding studies, our research discovered that Salvigenin hindered the activity of the PI3K/AKT/GSK-3β pathway, thus impeding HCC cell aerobic glycolysis and chemoresistance. However, the PI3K agonist 740Y-P inverted the anti-cancer function of Salvigenin in HCC.

## Conclusion

All in all, through network pharmacological analysis and experiments, we have probed the underlying mechanism of Salvigenin in HCC. As a result, Salvigenin may dampen HCC cell proliferation, migration, and invasion and weaken cell glycolysis and chemoresistance mainly by modulating the PI3K/AKT/GSK-3β pathway. Nonetheless, our study still has some limitations. In more animal studies, we will confirm the function and molecular mechanism of Salvigenin in the context of HCC.

## Supplementary Information

Supplementary Figure 1 The profiles of PIK3CA, AKT1, and GSK3B in HCC tissues and adjacent normal tissues. A-C: The profiles of PIK3CA, AKT1, and GSK3B in HCC tissues and adjacent normal tissues were determined through the GEPIA (http://gepia.cancer-pku.cn/) database. D-G: The Human Protein Atlas (https: //www. proteinatlas.org/) was adopted to check the profiles of PIK3CA, AKT1, and GSK3B proteins in HCC tissues and normal liver tissues. (PNG 2290 kb)

High resolution image (TIF 8192 kb)

Supplementary Figure 2 The correlation between the expression levels of PIK3CA, AKT1, and GSK3B and prognosis in HCC. A-C: The GEPIA database (http://gepia.cancer-pku.cn/) was introduced to verify the relationship between the levels of PIK3CA, AKT1, and GSK3B and the OS and RFS rates of HCC patients. (PNG 495 kb)

High resolution image (TIF 1670 kb)

Supplementary Figure 3 The toxicity of Salvigenin in tumor-bearing mice. A-B: ELISA ascertained ALT, AST, Scr, and BUN levels in the mouse serum. C: HE staining monitored pathological alterations in the heart, liver, spleen, lung, and kidney tissues of mice. (PNG 4175 kb)

High resolution image (TIF 14644 kb)

## Data Availability

The data sets used and analyzed during the current study are available from the corresponding author upon reasonable request.

## References

[1] Chidambaranathan-Reghupaty S, Fisher PB, Sarkar D (2021). Hepatocellular carcinoma (HCC): Epidemiology, etiology and molecular classification. Advances in Cancer Research.

[2] Lee M, Ko H, Yun M (2018). Cancer metabolism as a mechanism of treatment resistance and potential therapeutic target in hepatocellular carcinoma. Yonsei Medical Journal.

[3] Nguyen VC, Nguyen TH, Phan TH (2023). Fragment length profiles of cancer mutations enhance detection of circulating tumor DNA in patients with early-stage hepatocellular carcinoma. BMC Cancer.

[4] Zeng ZM, Mo N, Zeng J (2022). Advances in postoperative adjuvant therapy for primary liver cancer. World Journal of Gastrointestinal Oncology.

[5] Kim DW, Talati C, Kim R (2017). Hepatocellular carcinoma (HCC): Beyond sorafenib-chemotherapy. Journal of Gastrointestinal Oncology.

[6] Zheng R, Qu C, Zhang S, Zeng H, Sun K, Gu X, Xia C, Yang Z, Li H, Wei W, Chen W, He J (2018). Liver cancer incidence and mortality in China: Temporal trends and projections to 2030. Chinese Journal of Cancer Research.

[7] Xiao Z, Chen Z, Han R, Lu L, Li Z, Lin J, Hu L, Huang X, Lin L (2021). Comprehensive TCM treatments combined with chemotherapy for advanced non-small cell lung cancer: A randomized, controlled trial. Medicine (Baltimore).

[8] Wang S, Long S, Deng Z, Wu W (2020). Positive role of Chinese herbal medicine in cancer immune regulation. The American Journal of Chinese Medicine.

[9] Xu FQ, Dong MM, Wang ZF, Cao LD (2023). Metabolic rearrangements and intratumoral heterogeneity for immune response in hepatocellular carcinoma. Frontiers in Immunology.

[10] Jung J, Park S, Jang Y (2022). Clinical significance of glycolytic metabolic activity in hepatocellular carcinoma. Cancers.

[11] Feng J, Li J, Wu L, Yu Q, Ji J, Wu J, Dai W, Guo C (2020). Emerging roles and the regulation of aerobic glycolysis in hepatocellular carcinoma. Journal of Experimental & Clinical Cancer Research.

[12] Chen Q, Bao L, Huang Y, Lv L, Zhang G, Chen Y (2022). Clinical significance and immunogenomic landscape analysis of glycolysis-associated prognostic model to guide clinical therapy in hepatocellular carcinoma. Journal of Gastrointestinal Oncology.

[13] Yang X, Yang Y, Tang S, Tang H, Yang G, Xu Q, Wu J (2014). Anti-tumor effect of polysaccharides from Scutellaria barbata D. Don on the 95-D xenograft model via inhibition of the C-met pathway. Journal of Pharmacological Sciences.

[14] Ye CL, Huang Q (2012). Extraction of polysaccharides from herbal Scutellaria barbata D. Don (Ban-Zhi-Lian) and their antioxidant activity. Carbohydrate Polymers.

[15] Dai ZJ, Lu WF, Gao J, Kang HF, Ma YG, Zhang SQ, Diao Y, Lin S, Wang XJ, Wu WY (2013). Anti-angiogenic effect of the total flavonoids in Scutellaria barbata D. Don. BMC Complementary and Alternative Medicine.

[16] Dai Z, Liu X, Ji Z, Liu L, Kang H, Wang X, Diao Y (2008). The effect-enhancing and toxicity-reducing action of the extract of herba Scutellariae barbatae for chemotherapy in hepatoma H22 tumor-bearing mice. Journal of Traditional Chinese Medicine.

[17] Yin X, Zhou J, Jie C, Xing D, Zhang Y (2004). Anticancer activity and mechanism of Scutellaria barbata extract on human lung cancer cell line A549. Life Sciences.

[18] Choi BB, Choi JH, Park SR, Kim JY, Hong JW, Kim GC (2015). Scutellariae radix induces apoptosis in chemoresistant SCC-25 human tongue squamous carcinoma cells. The American Journal of Chinese Medicine.

[19] Wang Y, Yin S, Zhou Y, Zhou W, Chen T, Wu Q, Zhou L, Zheng S (2019). Dual-function of Baicalin in nsPEFs-treated hepatocytes and hepatocellular carcinoma cells for different death pathway and mitochondrial response. International Journal of Medical Sciences.

[20] Serino E, Chahardoli A, Badolati N, Sirignano C, Jalilian F, Mojarrab M, Farhangi Z, Rigano D, Stornaiuolo M, Shokoohinia Y, Taglialatela-Scafati O (2021). Salvigenin, a trimethoxylated flavone from Achillea wilhelmsii C. Koch, exerts combined lipid-lowering and mitochondrial stimulatory effects. Antioxidants.

[21] Namazi Sarvestani N, Sepehri H, Delphi L, Moridi FM (2018). Eupatorin and salvigenin potentiate doxorubicin-induced apoptosis and cell cycle arrest in HT-29 and SW948 human colon cancer cells. Asian Pacific Journal of Cancer Prevention.

[22] Noori S, Hassan ZM, Yaghmaei B, Dolatkhah M (2013). Antitumor and immunomodulatory effects of salvigenin on tumor bearing mice. Cellular Immunology.

[23] Yuanyuan, S., Qiong, B., Hongxun, T., & Xu, B. (2023). Application prospect of traditional Chinese medicine network pharmacology in food science research [published online ahead of print, 2023 Mar 7]. *Journal of the Science of Food and Agriculture*. 10.1002/jsfa.12541 10.1002/jsfa.12541 36882903

[24] Zhao L, Zhang H, Li N (2023). Network pharmacology, a promising approach to reveal the pharmacology mechanism of Chinese medicine formula [published online ahead of print, 2023 Feb 27]. Journal of Ethnopharmacology.

[25] Tian LY, Smit DJ, Jücker M (2023). The role of PI3K/AKT/mTOR signaling in hepatocellular carcinoma metabolism. International Journal of Molecular Sciences.

[26] Bai C, Zhao J, Su J (2022). Curcumin induces mitochondrial apoptosis in human hepatoma cells through BCLAF1-mediated modulation of PI3K/AKT/GSK-3β signaling. Life Sciences.

[27] Abdel-Rafei MK, Thabet NM, Rashed LA, Moustafa EM (2021). Canagliflozin, a SGLT-2 inhibitor, relieves ER stress, modulates autophagy and induces apoptosis in irradiated HepG2 cells: Signal transduction between PI3K/AKT/GSK-3β/mTOR and Wnt/β-catenin pathways; in vitro. Journal of Cancer Research and Therapeutics.

[28] Daina A, Michielin O, Zoete V (2019). SwissTargetPrediction: Updated data and new features for efficient prediction of protein targets of small molecules. Nucleic Acids Research.

[29] Xiao C, Liu S, Ge G (2023). Roles of hypoxia-inducible factor in hepatocellular carcinoma under local ablation therapies. Frontiers in Pharmacology.

[30] Wu Q, Chen Z, Ding Y, Tang Y, Cheng Y (2022). Protective effect of traditional Chinese medicine on non-alcoholic fatty liver disease and liver cancer by targeting ferroptosis. Frontiers in Nutrition.

[31] Llovet JM, Villanueva A, Lachenmayer A, Finn RS (2015). Advances in targeted therapies for hepatocellular carcinoma in the genomic era. Nature Reviews. Clinical Oncology.

[32] Wang, Y., & Deng, B. (2023). Hepatocellular carcinoma: Molecular mechanism, targeted therapy, and biomarkers. *Cancer Metastasis Reviews*. 10.1007/s10555-023-10084-4 10.1007/s10555-023-10084-4 36729264

[33] DeWaal D, Nogueira V, Terry AR, Patra KC, Jeon SM, Guzman G, Au J, Long CP, Antoniewicz MR, Hay N (2018). Hexokinase-2 depletion inhibits glycolysis and induces oxidative phosphorylation in hepatocellular carcinoma and sensitizes to metformin. Nature Communications.

[34] Hu L, Zeng Z, Xia Q, Liu Z, Feng X, Chen J, Huang M, Chen L, Fang Z, Liu Q, Zeng H, Zhou X, Liu J (2019). Metformin attenuates hepatoma cell proliferation by decreasing glycolytic flux through the HIF-1α/PFKFB3/PFK1 pathway. Life Sciences.

[35] Lv L, Xu YP, Zhao D, Li FL, Wang W, Sasaki N, Jiang Y, Zhou X, Li TT, Guan KL, Lei QY, Xiong Y (2013). Mitogenic and oncogenic stimulation of K433 acetylation promotes PKM2 protein kinase activity and nuclear localization. Molecular Cell.

[36] Lien EC, Dibble CC, Toker A (2017). PI3K signaling in cancer: Beyond AKT. Current Opinion in Cell Biology.

[37] Zhang M, Zhang H, Hong H, Zhang Z (2019). MiR-374b re-sensitizes hepatocellular carcinoma cells to sorafenib therapy by antagonizing PKM2-mediated glycolysis pathway. American Journal of Cancer Research.

[38] Hashemi M, Etemad S, Rezaei S (2023). Progress in targeting PTEN/PI3K/Akt axis in glioblastoma therapy: Revisiting molecular interactions. Biomedicine & Pharmacotherapy.

[39] Langdon CG (2023). Nuclear PTEN’s functions in suppressing tumorigenesis: Implications for rare cancers. Biomolecules..

[40] Martini M, De Santis MC, Braccini L, Gulluni F, Hirsch E (2014). PI3K/AKT signaling pathway and cancer: An updated review. Annals of Medicine.

[41] Vanhaesebroeck B, Waterfield MD (1999). Signaling by distinct classes of phosphoinositide 3-kinases. Experimental Cell Research.

[42] Shariati M, Meric-Bernstam F (2019). Targeting AKT for cancer therapy. Expert Opinion on Investigational Drugs.

[43] Sheng T, Mao XB, Zhang SH (2020). CaMKKβ regulates proliferation, apoptosis, and glycolysis of hepatocellular carcinoma via PI3K/AKT pathway. Annals of Palliative Medicine.

[44] Liang MH, Chuang DM (2007). Regulation and function of glycogen synthase kinase-3 isoforms in neuronal survival. The Journal of Biological Chemistry.

[45] Wu X, Liu C, Li Z, Gai C, Ding D, Chen W, Hao F, Li W (2020). Regulation of GSK3β/Nrf2 signaling pathway modulated erastin-induced ferroptosis in breast cancer. Molecular and Cellular Biochemistry.

[46] Zhang S, Gao W, Tang J, Zhang H, Zhou Y, Liu J, Chen K, Liu F, Li W, Top SK, Wong AST, Zhang XK, Zhou H, Zeng JZ (2020). The roles of GSK-3β in regulation of retinoid signaling and sorafenib treatment response in hepatocellular carcinoma. Theranostics..

[47] Fang G, Zhang P, Liu J, Zhang X, Zhu X, Li R, Wang H (2019). Inhibition of GSK-3β activity suppresses HCC malignant phenotype by inhibiting glycolysis via activating AMPK/mTOR signaling. Cancer Letters.

