# Supplementary figures and images for: Salvigenin Suppresses Hepatocellular Carcinoma Glycolysis and Chemoresistance Through Inactivating the PI3K/AKT/GSK-3β Pathway
Source: Appl Biochem Biotechnol. 2023 May 2;195(8):5217–37. doi: 10.1007/s12010-023-04511-z (PMC10354167; doi:10.1007/s12010-023-04511-z)

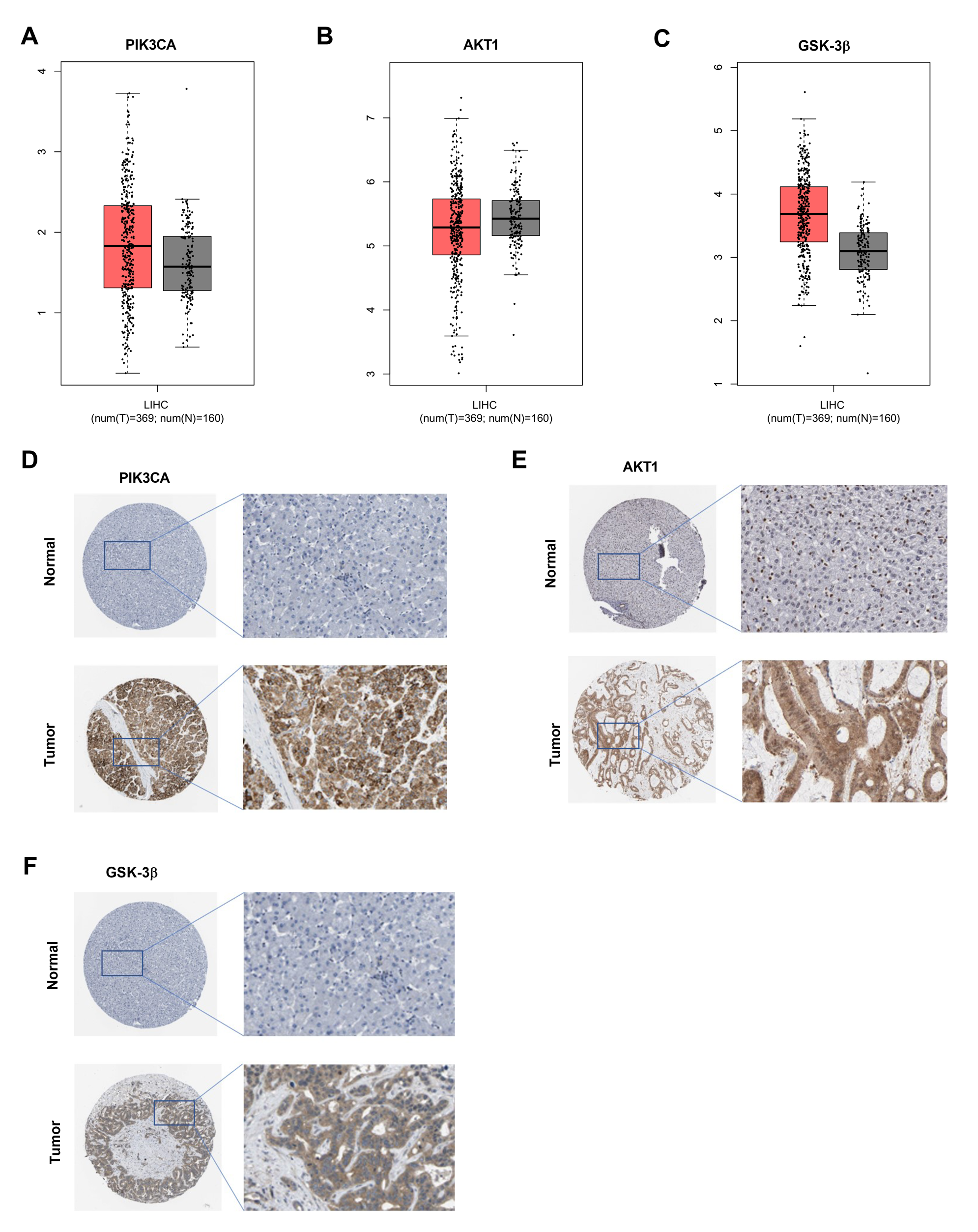

Supplement: Supplementary file 1 — The profiles of PIK3CA, AKT1, and GSK3B in HCC tissues and adjacent normal tissues. A-C: The profiles of PIK3CA, AKT1, and GSK3B in HCC tissues and adjacent normal tissues were determined through the GEPIA (http://gepia.cancer-pku.cn/) database. D-G: The Human Protein Atlas (https: //www. proteinatlas.org/) was adopted to check the profiles of PIK3CA, AKT1, and GSK3B proteins in HCC tissues and normal liver tissues. (PNG 2290 kb) [file 12010_2023_4511_Fig1_ESM.png]

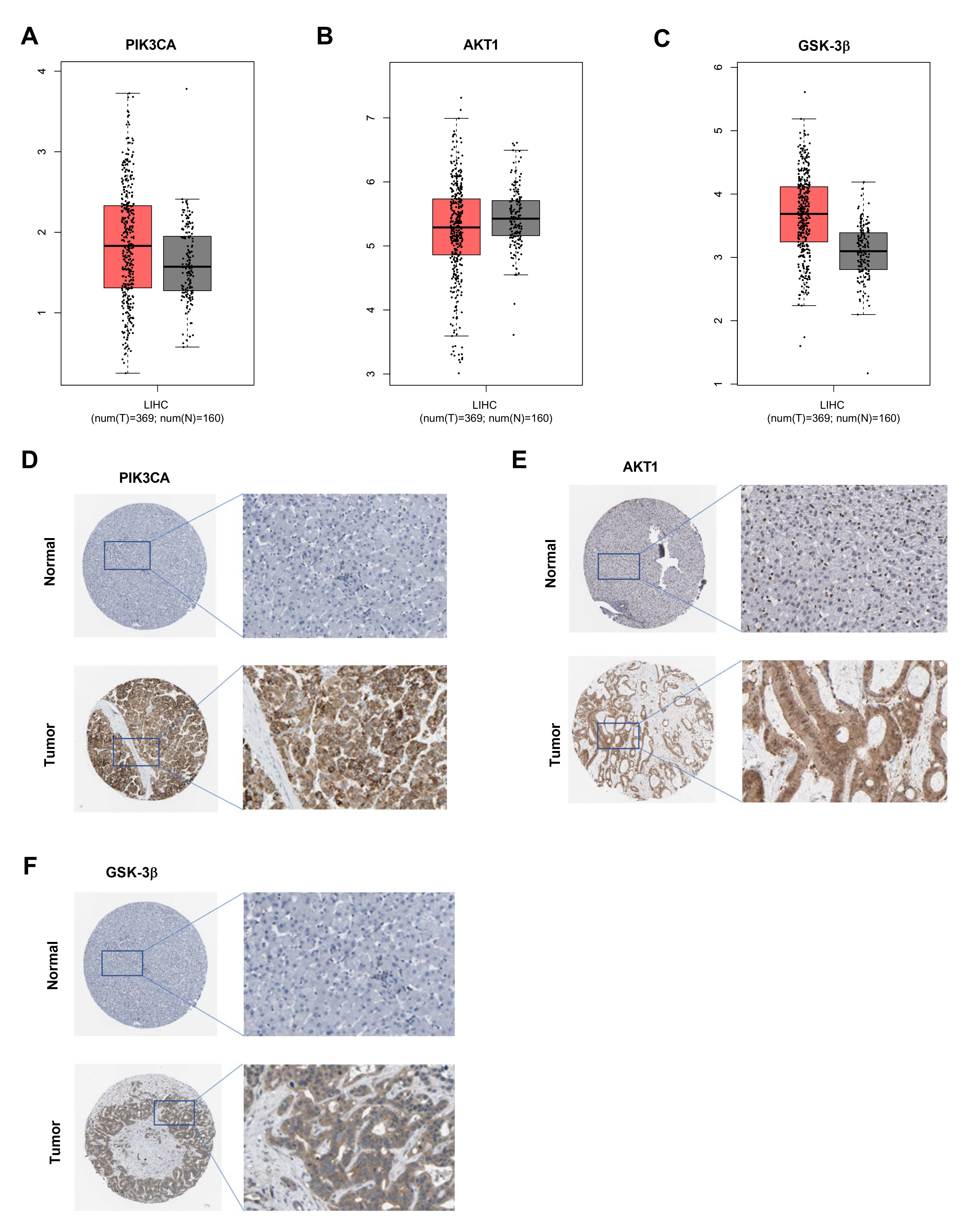

Supplement: Supplementary file 2 — High resolution image (TIF 8192 kb) [file 12010_2023_4511_MOESM1_ESM.tif]

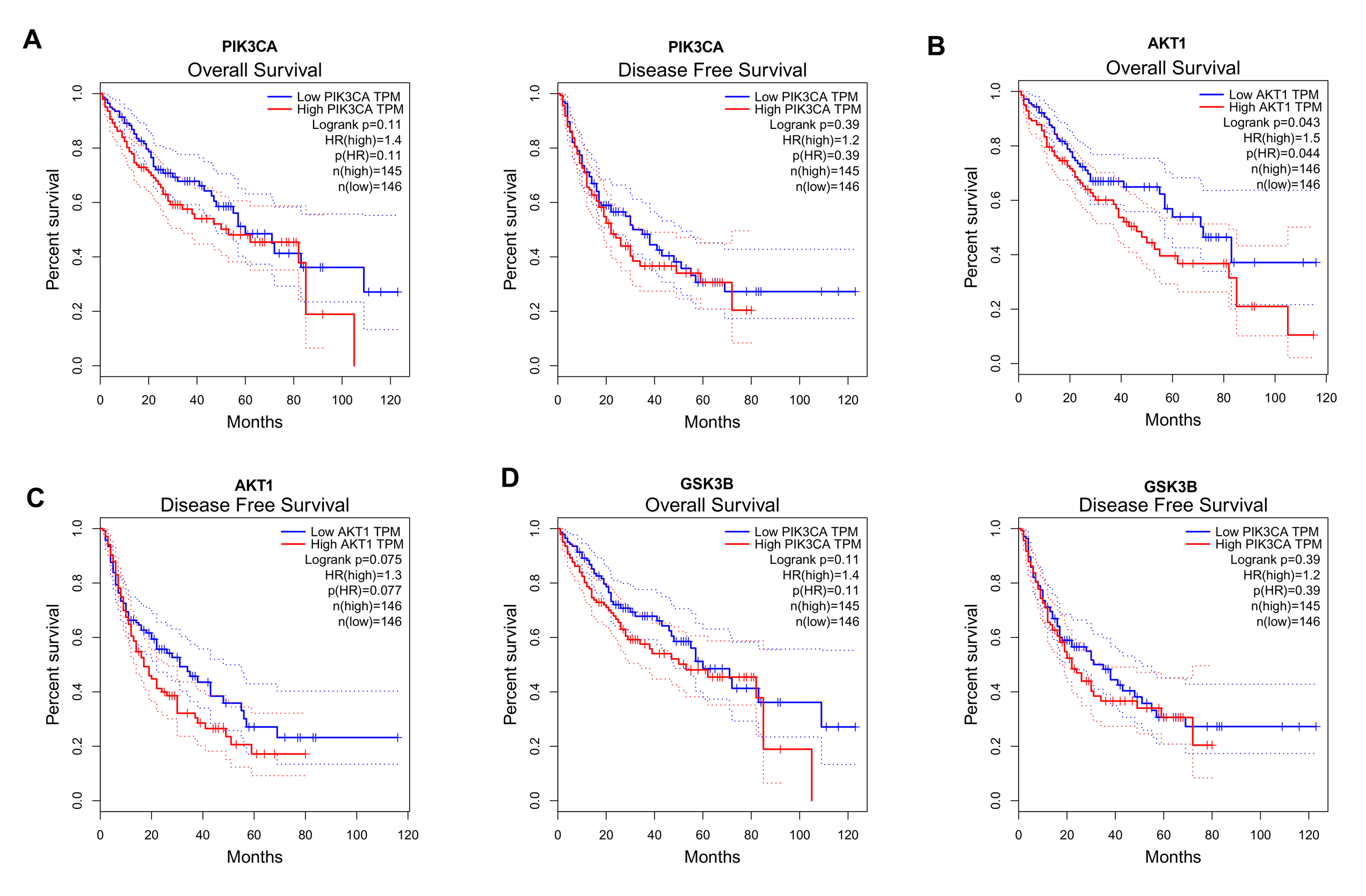

Supplement: Supplementary file 3 — The correlation between the expression levels of PIK3CA, AKT1, and GSK3B and prognosis in HCC. A-C: The GEPIA database (http://gepia.cancer-pku.cn/) was introduced to verify the relationship between the levels of PIK3CA, AKT1, and GSK3B and the OS and RFS rates of HCC patients. (PNG 495 kb) [file 12010_2023_4511_Fig2_ESM.png]

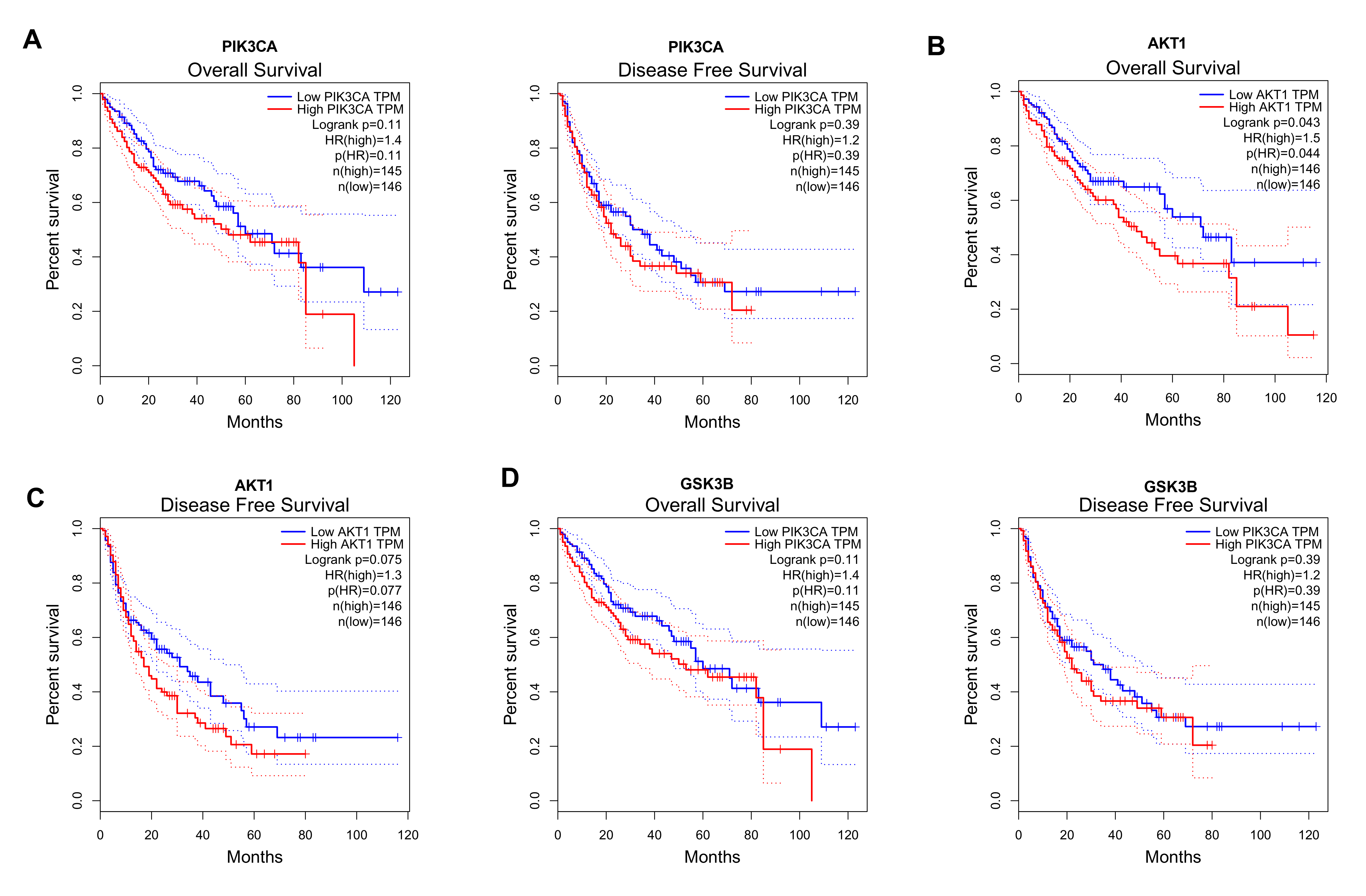

Supplement: Supplementary file 4 — High resolution image (TIF 1670 kb) [file 12010_2023_4511_MOESM2_ESM.tif]

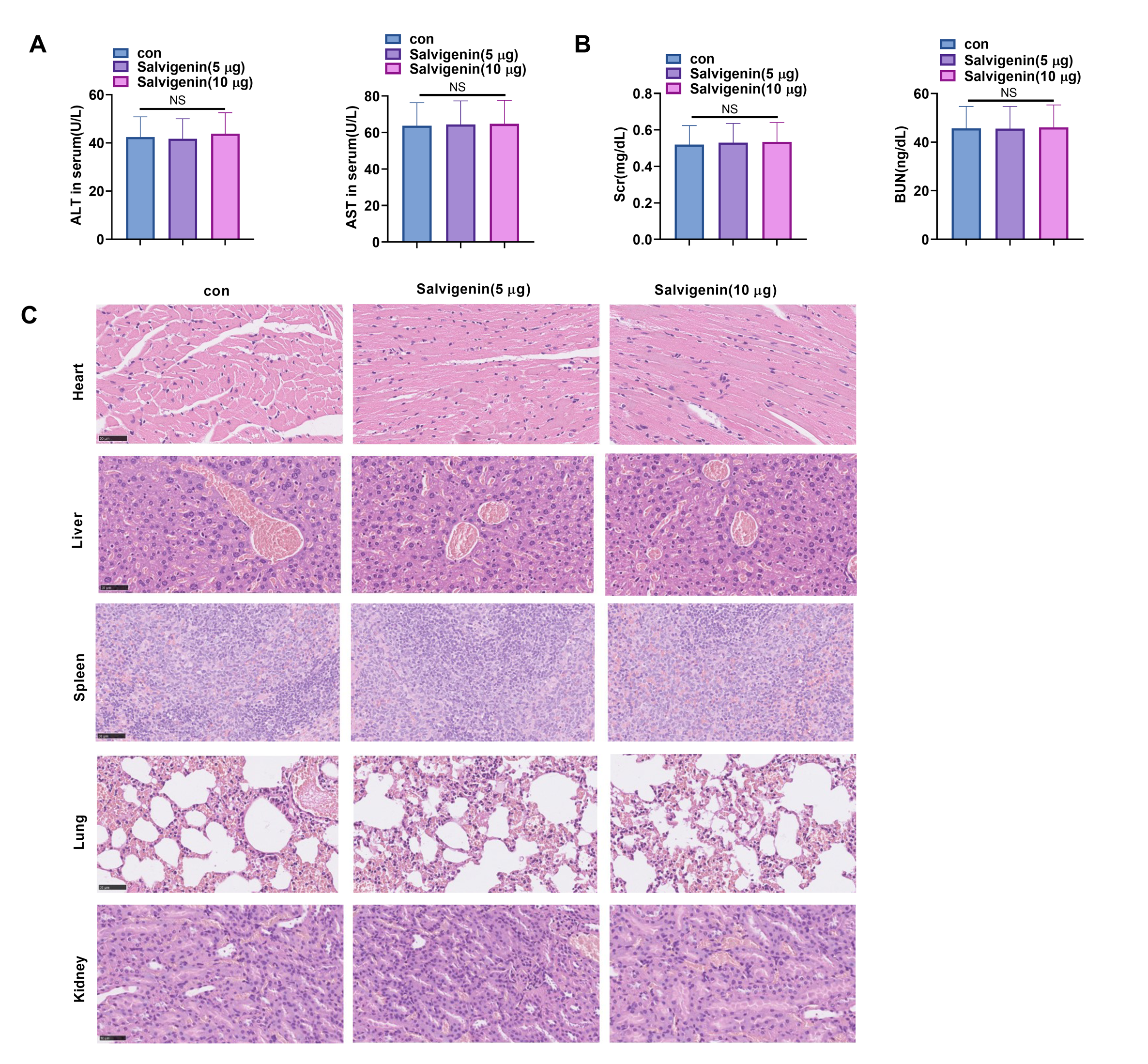

Supplement: Supplementary file 5 — The toxicity of Salvigenin in tumor-bearing mice. A-B: ELISA ascertained ALT, AST, Scr, and BUN levels in the mouse serum. C: HE staining monitored pathological alterations in the heart, liver, spleen, lung, and kidney tissues of mice. (PNG 4175 kb) [file 12010_2023_4511_Fig3_ESM.png]

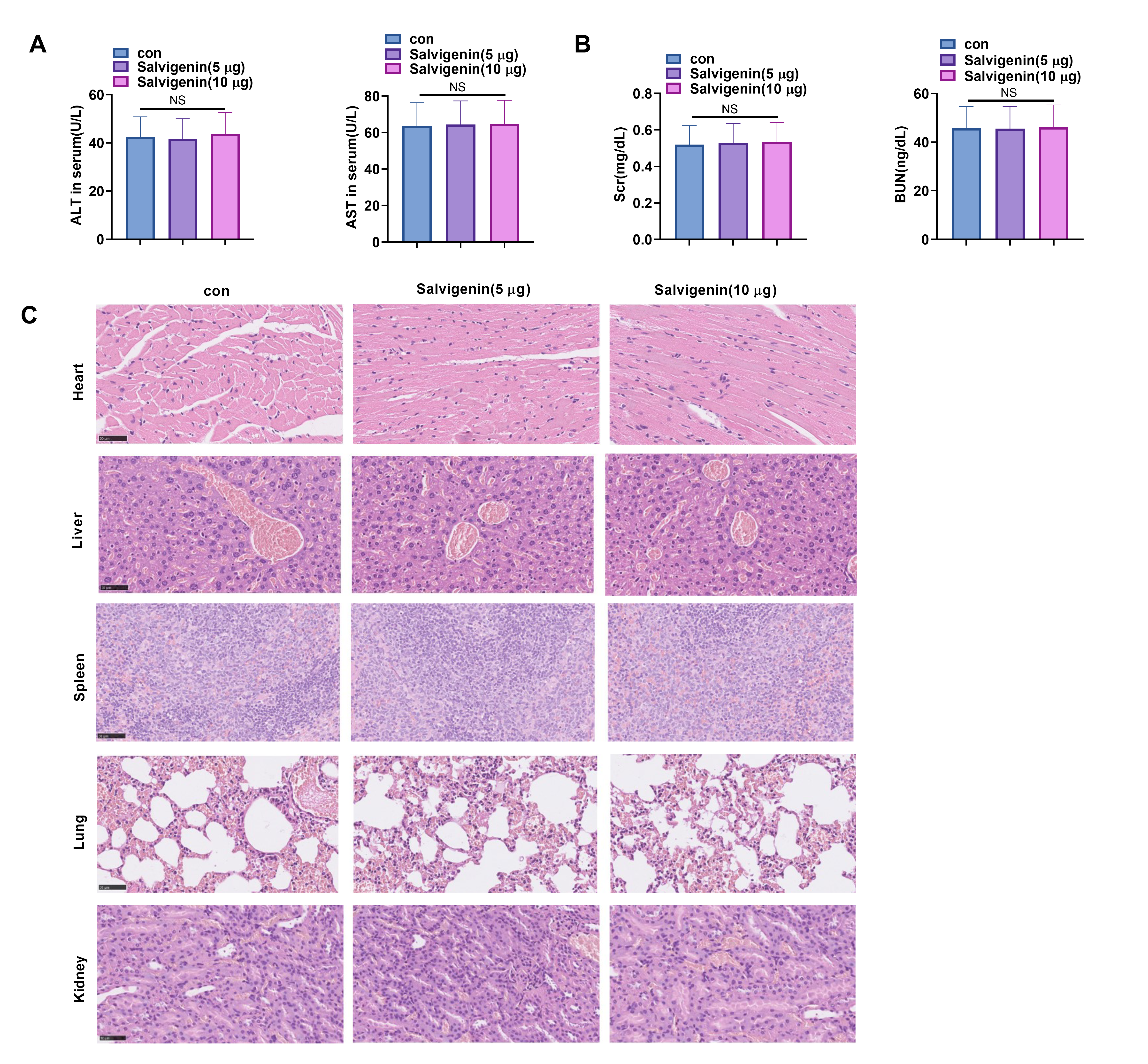

Supplement: Supplementary file 6 — High resolution image (TIF 14644 kb) [file 12010_2023_4511_MOESM3_ESM.tif]
